# RNase P cleavage of pseudoknot substrates reveals differences in active site architecture that depend on residue N-1 in the 5’ leader

**DOI:** 10.1080/15476286.2024.2427906

**Published:** 2025-01-20

**Authors:** David M. Kosek, J. Luis Leal, Ema Kikovska-Stojanovska, Guanzhong Mao, Shiying Wu, Samuel C. Flores, Leif A. Kirsebom

**Affiliations:** a Department of Cell and Molecular Biology, Biomedical Centre, Uppsala University, Uppsala, Sweden; b Department of Medical Biochemistry and Microbiology, Biomedical Centre, Uppsala University, Uppsala, Sweden; c Department of Ecology and Genetics, Evolutionary Biology Center EBC, Uppsala University, Uppsala, Sweden; d Merck Healthcare KGaA, Global Regulatory CMC & Devices, Darmstadt, Germany; e Bio-Works AB, Uppsala, Sweden; f Department of Animal Biosciences, Swedish University of Agricultural Sciences, Uppsala, Sweden; g Department of Biochemistry and Biophysics, Stockholm University, Solna, Sweden

**Keywords:** RNase P, ribozyme, divalent metal ions, model substrates, tRNA processing

## Abstract

We show that a small biotin-binding RNA aptamer that folds into a pseudoknot structure acts as a substrate for bacterial RNase P RNA (RPR) with and without the RNase P C5 protein. Cleavage in the single-stranded region in loop 1 was shown to depend on the presence of a RCCA-motif at the 3’ end of the substrate. The nucleobase and the 2’hydroxyl at the position immediately 5’ of the cleavage site contribute to both cleavage efficiency and site selection, where C at this position induces significant cleavage at an alternative site, one base upstream of the main cleavage site. The frequencies of cleavage at these two sites and Mg^2+^ binding change upon altering the structural topology in the vicinity of the cleavage site as well as by replacing Mg^2+^ with other divalent metal ions. Modelling studies of RPR in complex with the pseudoknot substrates suggest alternative structural topologies for cleavage at the main and the alternative site and a shift in positioning of Mg^2+^ that activates the H_2_O nucleophile. Together, our data are consistent with a model where the organization of the active site structure and positioning of Mg^2+^ is influenced by the identities of residues at and in the vicinity of the site of cleavage.

## Introduction

The endoribonuclease P, RNase P, generates tRNAs with a single phosphate at their 5’ termini. RNase P is composed of one RNA (RNase P RNA or RPR) moiety, while the number of protein subunits varies depending on origin: one protein in bacteria (in *Escherichia coli* referred to as the C5 protein), four to five in Archaea and nine to ten in Eukarya [1]. Irrespective of origin, the catalytic activity resides in the RPR [2–5]; however, RNase P activity solely composed of protein(s) exist as well [6–10].

Apart from being involved in tRNA processing, RNase P also processes several other natural RNAs, including mRNAs and riboswitches, the precursors to 4.5S RNA and tmRNA, synthetic model hairpin substrates, phage RNAs and small single-stranded RNAs [11–25]. Moreover, in RNA plant viruses such as the tobacco mosaic virus (TMV) and tobacco yellow mosaic virus (TYMV), the RNA 3’ termini fold into a tRNA-like structure that ends with CCA. Folding of these structures depends on formation of a pseudoknot and these tRNA-like structures also act as RNase P substrates [26–30]. Pseudoknot structural elements are also present in mRNAs, where they can influence the reading frame during translation [31,32]. Whether pseudoknots are targeted by RNase P *in vivo* remains an open question (see discussion). However, we emphasize that understanding factors that influence the processing of various RNA substrates, including pre-tRNAs and model substrates such as pseudoknot structures, is important to dissect and conceive the molecular features that determine efficient and correct RNase P processing.

The residue positioned 5’ of the scissile bond (referred to as N_−1_ in e.g. precursor-tRNA) plays an important role in the processing of various substrates by RNase P, see e.g. [23, 33–40]. This has also been discussed to be the case for pseudoknot structures [30]. The structures of different pseudoknots have been studied in detail both by X-ray crystallography and nuclear magnetic resonance spectroscopy, NMR [41–47; for a review see 48]. Of these, the biotin-binding RNA aptamer is a pseudoknot structure of the hairpin (H-) type, and the crystal structure was solved at 1.3 Å resolution [49]. The pseudoknot in the TYMV tRNA-like structure, which was recently solved at 2.0 Å resolution [47], is another H-type pseudoknot. Since the TYMV tRNA-like structure acts as a substrate for RNase P [28–30], these structures are suited as experimental models towards understanding factors and the structure–function relation in cleavage mediated by RNase P and its RNA component, RPR.

The crystal structure of bacterial RNase P in complex with tRNA is available and represents a post-cleavage structure [50], and recently the cryo-EM structure of bacterial RNase P in complex with pre-tRNA was reported [51]. Albeit our understanding of the interaction between the 5’ leader of the substrate and RNase P is increasing, the interaction between the RPR and residue N_−1_ in different substrates is limited. This is in particular true for how non-conventional substrates interact with the RPR. Given that *E. coli* (*Eco*) RPR with and without the C5 protein cleaves RNA pseudoknot substrates [28–30] we were interested in understanding whether the biotin RNA aptamer, for which a high-resolution structure is available [49], can act as substrate for *Eco* RPR. If so, study cleavage as a function of the nucleobase identity immediately 5’ of the cleavage site (N_−1_ in pre-tRNAs) and its influence on the Mg^2+^ requirement, and use this information and available structures in modelling studies to extract information about the architecture at and in the vicinity of the cleavage site.

Here, we provide data showing that the biotin RNA aptamer [49] tagged with a 3’ GCCAC trailer is cleaved by both bacterial (with and without the C5 protein) and archaeal RPR. The cleavage efficiency was, however, substantially lower compared to cleavage of other substrates such as model hairpin substrates [23]. Moreover, the identity of the nucleobase 5’ of the cleavage site (‘N_−1_’) influences cleavage differently with respect to both site selection and efficiency of cleavage compared to cleavage of model hairpin substrates. Changing base pairs in the substrate in the vicinity of the cleavage site as well as in the RPR influenced site selection and Mg^2+^ binding. The data, complemented with dynamical modelling using MacroMoleculeBuilder (MMB), provided a rationale to the observed variation in site selection in response to changing the ‘N_−1_’ identity and its influence on Mg^2+^ requirement. Together, our data are consistent with a model where the structural architecture of the active site depends on the positioning of substrate residues and Mg^2+^ at, and in the vicinity of, the cleavage site. Finally, we foresee that our findings will enhance our understanding of RNase P processing of RNA molecules that contain pseudoknot structures such as mRNA *in vivo*.

## Materials and methods

### Preparation of substrates, RPRs and C5 protein

Substrates were purchased from Dharmacon (USA) and IBA GmbH (Germany) and purified on a 15% (w/v) denaturing PAGE gel followed by Bio-Trap extraction overnight (Schleicher and Schuell, GmbH, Germany; Elutrap in USA and Canada) and phenol-chloroform extraction. The different substrates were 5’ end-labelled with [γ-^32^P]-ATP and gel-purified as previously described [22,23].

The *Eco* RPR, *HyoP* RPR and *Pfu* RPR were generated as run-off transcripts using T7 DNA-dependent RNA polymerase and PCR-amplified templates [23, 52–54].

The *E. coli* C5 protein derivative, His6-C5, was purified as described elsewhere [23,55].

### Biotin affinity chromatography

In the biotin-binding assays, 0.5 µg of RNA substrate in buffer D [50 mM Tris-HCl (pH 7.5), 100 mM NH_4_Cl, 10 mM MgCl_2_] was applied on biotin agarose columns [prepared by adding 20 µL biotin agarose to Pierce Micro-Spin Columns (Thermo Fisher, number 89879) and packed in microcentrifuge at 100 × g]. The column was washed with five column volumes of buffer D. Bound RNA was eluted with five column volumes of buffer D containing 5 mM biotin. Following ethanol precipitation and re-suspension in double distilled water, the eluted RNA was quantified by measuring the absorbance at 260 nm using a NanoDrop.

### Assay conditions

RPR-mediated cleavage without the C5 protein was conducted at 37°C in buffer C [50 mM MES (final pH 6.1), 0.8 M NH_4_OAc] with indicated Mg(OAc)_2_ concentrations. The RPR was pre-incubated in buffer C and Mg(OAc)_2_ for 10 min to allow for folding into the active conformation, after which prewarmed (37°C) substrate was added. All the experiments in the absence of C5 were conducted under single turn over conditions with ≤0.02 μM substrate. For the concentrations of *Eco* RPR, see table and figure legends.

Reactions with His6-C5 protein were carried out in buffer A [50 mM Tris-HCl, pH 7.2 (final), 100 mM NH_4_Cl] supplemented with 10 mM MgCl_2_. *Eco* RPR was pre-incubated in buffer A at 37°C for 10 min. The purified His6-C5 was added, followed by the addition of prewarmed (37°C) substrate and incubation for 30 min at 37°C. The concentrations of *Eco* RPR and His6-C5 were 0.004 μM and 0.21 μM, respectively, and the concentration of substrate was ≤0.02 μM.

All reactions were terminated by the addition of two volumes of stop solution (10 M urea, 100 mM EDTA). Products were separated on 25% (w/v) denaturing polyacrylamide/7 M urea gels.

### Determination of kinetic constants under single turnover conditions

The kinetic constants k_obs_ and k_obs_/K^sto^ (=k_cat_/K_m_) were determined under saturating single turnover conditions in buffer C (pH 6.1) and 800 mM Mg(OAc)_2_ as described elsewhere, see e.g. [22,25]. The final substrate concentration was ≤0.02 μM, while the *Eco* RPR concentration varied between 0.4 and 26 μM, depending on RPR-substrate combination. For calculations, the 5’ cleavage fragments were used to measure product formation. Incubation times were adjusted to ensure that the velocity measurements were in the linear range (≤40% of the substrate consumed). The k_obs_ and k_obs_/K^sto^ constants were obtained by linear regression from Eadie-Hofstee plots. The K^sto^ (≈K_d_ [23]) constants were calculated using k_obs_ and k_obs_/K^sto^ values.

### Substrate digestion with nuclease P1

Nuclease P1 was purchased from Sigma Aldrich (USA) and prepared according to company instructions. The RNA substrate was denatured at 95°C for 5 min and then added to buffer P1 (40 mM NaOAc, pH 5.3, 0.5 mM ZnSO_4_) prewarmed to 70°C. This was immediately followed by the addition of one ng (corresponding to ≥0.0002 units) nuclease P1 (Sigma Aldrich), to a final reaction volume of 10 μL and incubation at 70°C for 1 min. The reaction was terminated by the addition of double volume of stop solution (10 M urea, 100 mM EDTA).

### Modelling

MacroMoleculeBuilder, MMB (v. 2.19), is a general-purpose, multiscale, internal-coordinate macromolecular modelling code used for RNA modelling [56–58]. MMB is not a Molecular Dynamics (MD) package. MD works in cartesian coordinates whereas MMB works in internal coordinates, which eases implementation of integrative multiresolution modelling. However, internal coordinates greatly reduce the degrees of freedom of the system. Moreover, MMB uses water droplets, which are known to cause surface tension artefacts, unlike the water boxes typically used in MD. In earlier work, we showed that MMB and MD simulations are complementary, as MMB can create models that are easily sent on to MD for confirmation. Similarly, MMB can suggest tightly focused hypotheses for, or rationalization of, experimental results [59–61; see also below].

We used MMB to model the pseudoknot substrate in complex with RPR under solvation conditions. We used the crystal structure of RNase P in complex with tRNA (PDB code 3Q1R, resolution 4.2 Å [50]), replacing the tRNA with the biotin RNA aptamer crystal structure (PDB code 1F27, resolution 1.3 Å [49]). The Mg^2+^ concentration was set to one metal ion per 3.5 nucleotides (see e.g. [39] and references therein). These Mg^2+^ ions were initially placed well outside the cleavage site to prevent any biased results. The reported ion positions are the result of MMB dynamics, *i.e*. Coulomb and other interactions between ions, water, and RNA residues. The RPR-substrate complex was modelled independently for each variant (30 runs per variant).

## Results

### Importance of the N_9(−1)_ identity for Eco RPR cleavage of a pseudoknot with and without the C5 protein

**Figure 1.**
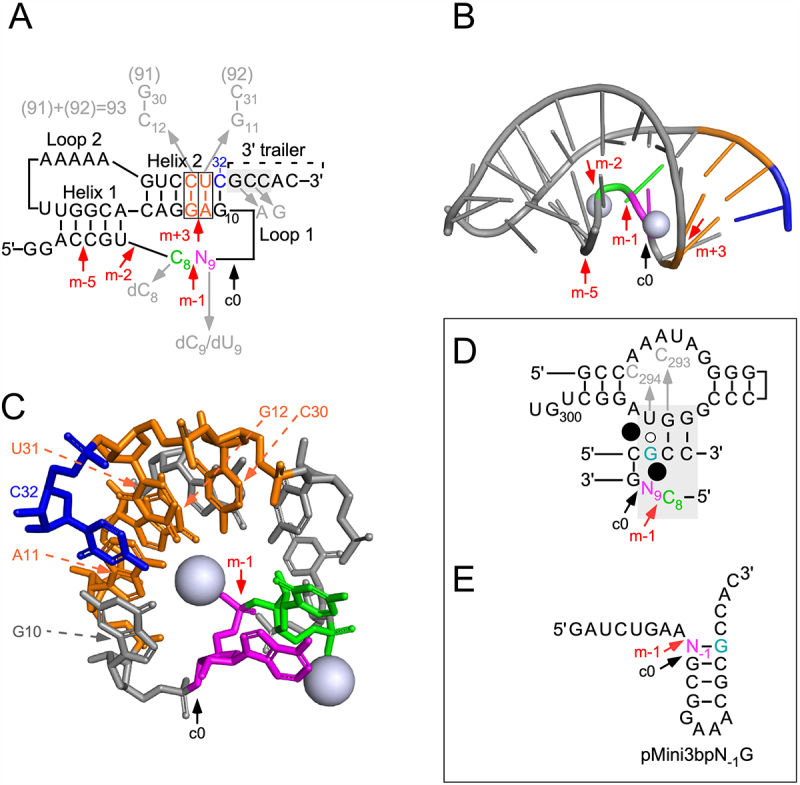
(A) Secondary structure of the pseudoknot (pPK) substrates used in the present study. Residue N_9_, which is an A in the original pseudoknot [49], is marked in magenta and was changed to C, G and U as indicated in the main text. The grey arrows mark the replacement of the 2’-OH with 2’-H at C_9_ (or U_9_) and C_8_ (highlighted in green), and replacement of nucleobases at indicated positions (marked in grey). The black arrow marks the c0 (canonical) cleavage site while the red arrows mark the alternative cleavage sites m-1, m-2 (see Figure 5), m+3 (cleavage at +3 was only detected using pPKdU_9_G_33_, see main text) and m-5 (see Figure 5, cleavage with *HyoP* RPR). Residues in the grey box mark residues that interact with the RPR, see Figure 1D. The 5’-GCCAC-3’ trailer was added as discussed in the main text. (B) The crystal structure of the pPK RNA biotin-binding aptamer at 1.3 Å, PDB 1F27 [49]. Residues discussed in the text are highlighted and coloured as in Figure 1A and 1C. The RPR cleavage sites are marked with arrows: c0 (canonical cleavage site) in black and the alternative cleavage sites m-1, m-2, m+3 and m-5 in red. The spheres represent Mg^2+^-ions. See also Figure 1A and 1C. (C) Part of the crystal structure of the original pPK RNA biotin-binding aptamer at 1.3 Å, PDB 1F27 [49]. Residues highlighted: A_9_ (magenta), C_8_ (green), the 3’ terminal C_32_ (blue), and A_11_-U_31_/G_12_-C_30_ (Orange). The spheres represent two Mg^2+^-ions and the arrows mark the cleavage sites c0 (black) and m-1 (red). (D) The RCCA-RNase P RNA interaction (interacting residues underlined and shown in the grey box) where the GCC sequence at the 3’ termini pairs with G_292_-G_293_-U_294_ in *Eco* RPR_wt_. N_9_, the residue immediately 5’ of cleavage at c0 (black arrow) is highlighted in magenta and the alternative cleavage site m-1 is marked with a red arrow. The black spheres represent Mg^2+^ ions positioned in the vicinity of the cleavage site. The arrows marked in grey indicate the substitutions introduced in *Eco* RPR, for details see main text. The residue corresponding to the tRNA discriminator base is coloured in turquoise, see also the main text. (E) Secondary structure of pMini3bpN_−1_G. The black arrow marks the main cleavage site c0 while the red arrow the alternative site m-1. Note that c0 and m-1 correspond to cleavage at +1 and −1 as previously reported, for details see e.g. Wu et al. [23]. The residue corresponding to the tRNA discriminator base is coloured in turquoise, see also the main text.

**Figure 2.**
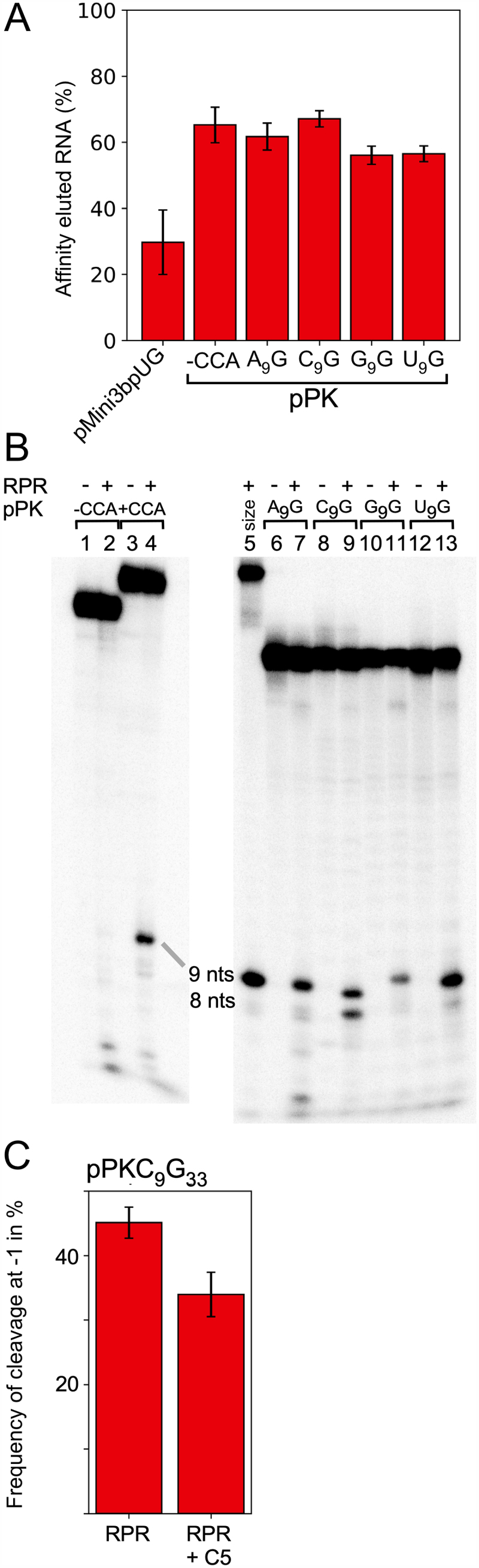
(A) Biotin binding. Amount of biotin-eluted pMini3bpUG and pPK RNA expressed as a percentage of total RNA applied to a biotin column (see Materials and Methods). The data represent mean ± experimental errors calculated from at least three independent experiments. (B) Cleavage of pPK variants with *Eco* RPR_wt_ in buffer C at 800 mM Mg(OAc)_2_ as indicated. The concentration of substrate was ≤0.02 μM while the *Eco* RPR_wt_ concentration was 6.4 μM (pPK) and 0.8 µM for the pATSerUG control (ctrl) in lane 5. Lane 1, pPK lacking the 5’-GCCAC 3’ trailer without RPR, incubation for 360 min; lane 2, pPK lacking the 5’-GCCAC 3’ trailer with RPR, incubation for 360 min; lane 3, pPK (pPKA_9_G_33_) with the 5’-GCCAC 3’ trailer without RPR, incubation for 90 min; lane 4, pPK (pPKA_9_G_33_) with the 5’-GCCAC 3’ trailer with RPR, incubation for 90 min; lane 5, pATSerUG (5’ leader size control) with RPR, incubation for 5 s; lane 6, pPKA_9_G_33_ without RPR, incubation for 90 min; lane 7, pPKA_9_G_33_ with RPR, incubation for 90 min; lane 8, pPKC_9_G_33_ without RPR, incubation for 60 min; lane 9, pPKC_9_G_33_ with RPR, incubation for 60 min; lane 10, pPKG_9_G_33_ without RPR, incubation for 90 min; lane 11, pPKG_9_G_33_ with RPR, incubation for 90 min; lane 12, pPKU_9_G_33_ without RPR, incubation for 60 min; lane 13, pPKU_9_G_33_ with RPR, incubation for 60 min. 9 and 8 nts mark the size of the 5’ cleavage product and the upper band the uncleaved substrate. (C) Frequency of cleavage of pPKC_9_G_33_ at m-1 with *Eco* RPR_wt_ in the absence and presence of C5. The frequency of cleavage at m-1 was determined as previously described [23,25] and represents the mean ± experimental errors of at least three independent experiments.

To understand whether the *in vitro* selected biotin-binding RNA aptamer can be cleaved by RNase P we generated pPK, which has the same sequence as reported by Nix et al. [49] (Figure 1A–C). pPK binds biotin suggesting that it acquired the expected fold. The unrelated RNA, pMini3bpUG, also binds biotin but with lower affinities (Figure 2A).

To test whether the pPK RNA is cleaved by *Eco* RPR_wt_ in the absence of the C5 protein we decided to perform the experiments discussed below at 800 mM Mg^2+^ (or as indicated). As we previously argued, at this Mg^2+^ concentration, the likelihood to detect cleavage increases. In addition, this would allow us to make comparison with previous findings using pre-tRNA and model hairpin substrates such as pMini3bp (Figure 1E) [23,25]. We also emphasize that in this study we focus on the RPR alone reaction since the C5 protein interacts with N_−4_ – N_−8_ in the pre-tRNA 5’ leader (the corresponding residues are part of helix 1 in the pPK substrates) but not with N_−1_ and N_−2_ [63], which correspond to N_9_ and N_8_ in the pPK RNA (Figure 1A).

When we exposed this RNA to *Eco* RPR_wt_, no apparent 5’ cleavage product could be detected (Figure 2B, lanes 1 and 2). This is not surprising since pPK lacks the 3’ RCC-motif that plays an important role in RPR-mediated cleavage (the RCCA-RPR interaction, interacting residues underlined, see also Figure 1D [64–68]). Hence, we designed a variant carrying the 5’-GCCAC trailer at the 3’ end and this RNA is referred to as pPKA_9_G_33_. The N_9_ residue corresponds to N_−1_ in pre-tRNAs and other model substrates and the underlined G (G_33_, see Figure 1A) corresponds to the discriminator base at position +73 in tRNA (see below; Figure 1A, D and E [69]). The pPKA_9_G_33_ binds biotin with similar affinity as the original RNA aptamer structure suggesting that the 5’-GCCAC trailer does not induce changes in the structure that affect the binding affinity for biotin (Figure 2A).

*Eco* RPR cleaved pPKA_9_G_33_ generating a 9-nt long 5’ cleavage product in the presence of 800 mM Mg^2+^ [25] and it co-migrated with the 5’ fragment generated by cleavage of the well-characterized pATSerUG model substrate (Figure 2B, lane 5). This suggested that the cleavage site was between residues A_9_ and G_10_ (see Figure 1A-C). The 5’ fragment also co-migrated with a nuclease P1 cleavage product suggesting that cleavage generated products with 5’-phosphate and 3’-hydroxyl groups at their ends as expected for RPR-mediated cleavage (*Supplemental Figure S1*; see e.g. [2,19]). We conclude that pPKA_9_G_33_ acts as substrate for *Eco* RPR_wt_ and it is cleaved mainly between residue A_9_ and G_10_. For comparison of choice of cleavage site using these substrates we hereafter refer to cleavage between positions 9 and 10 as ‘c0’ or the ‘canonical’ cleavage site, while cleavage between N_8_ (C_8_) and N_9_ is referred to as the ‘m-1’ site (see below and Figure 1A–C). We emphasize that c0 and m-1 correspond to the +1 and −1 cleavage sites assignments used in previous studies of pre-tRNAs and other model substrates (cf. Figure 1A and E) [25].

For site selection and cleavage efficiency, the identity of the N_−1_ residue (N_9_ in pPK) plays an important role in RPR-mediated cleavage of pre-tRNA and other model substrates (Figure 1E [23,25,39]). We therefore generated variants in which A_9_ was substituted with C_9_, G_9_ and U_9_ and these are referred to as pPKC_9_G_33_, pPKG_9_G_33_ and pPKU_9_G_33_, respectively (Figure 1A). These variants bind biotin with similar affinity as pPKA_9_G_33_ suggesting that the N_9_ identity does not affect biotin binding (Figure 2A). *Eco* RPR_wt_ cleaved these three RNAs at the c0 site but interestingly the C_9_ variant was also cleaved at the m-1 site (*i.e*. between N_8_ and N_9_; see above), with almost equal frequency as observed for cleavage at c0 (Figure 2B, lane 9; see also *Supplemental Figure S1*). As for pPKA_9_G_33_, these three N_9_ variants generate 5’ phosphate and 3’ hydroxyl groups at their ends as a result of cleavage with *Eco* RPR_wt_ (*Supplemental Figure S1*).

The *Eco* RPR_wt_ alone reaction requires high Mg^2+^ while addition of the C5 protein lowers the Mg^2+^ requirement [2]. Typically, cleavage in the presence of C5 is conducted at 10 mM Mg^2+^ (see Materials and Methods). Addition of C5 resulted in similar cleavage patterns compared to what we observed in its absence at 800 mM Mg^2+^. Even the C_9_ variant was cleaved both at the c0 and the m-1 sites albeit the frequency of cleavage at m-1 was slightly lower with C5 (Figure 2C). The A_9_, G_9_ and U_9_ variants were predominantly cleaved at the c0 site with and without the C5 protein (Figure 2B; data not shown for the A_9_, G_9_ and U_9_ variants in the presence of C5). In this context, the rate of cleavage of pPKC_9_G_33_ at c0 (0.24 ± 0.028%/min) and m-1 (0.16 ± 0.022%/min) with C5 is similar as in the absence of C5 at 800 mM Mg^2+^, c0 (0.19 ± 0.0045%/min) and m-1 (0.17 ± 0.022%/min). Thus, addition of the C5 protein lowers the Mg^2+^ requirement but does not appear to influence the rate of cleavage relative to cleavage in the RPR alone reaction at 800 mM Mg^2+^.

Taken together, the biotin-binding RNA aptamer tagged with the 5’-GCCAC trailer at the 3’ end acts as a substrate for *Eco* RPR with and without C5, and identity of the N_9_ residue (the residue immediately 5’ of the canonical cleavage, c0, site) plays a crucial role for site selection. In addition, despite that C_9_ most likely does not pair (Figure 1A–C) with any residue in the substrate, it promotes cleavage at the alternative site m-1. This is in contrast to what we expected on the basis of previous studies using pre-tRNAs and model hairpin substrates, where the absence of pairing between residue N_−1_ and the discriminator base result in cleavage mainly at the c0 (+1) site [see e.g. Refs 25,39].

### Cleavage as a function of divalent metal ions

**Figure 3.**
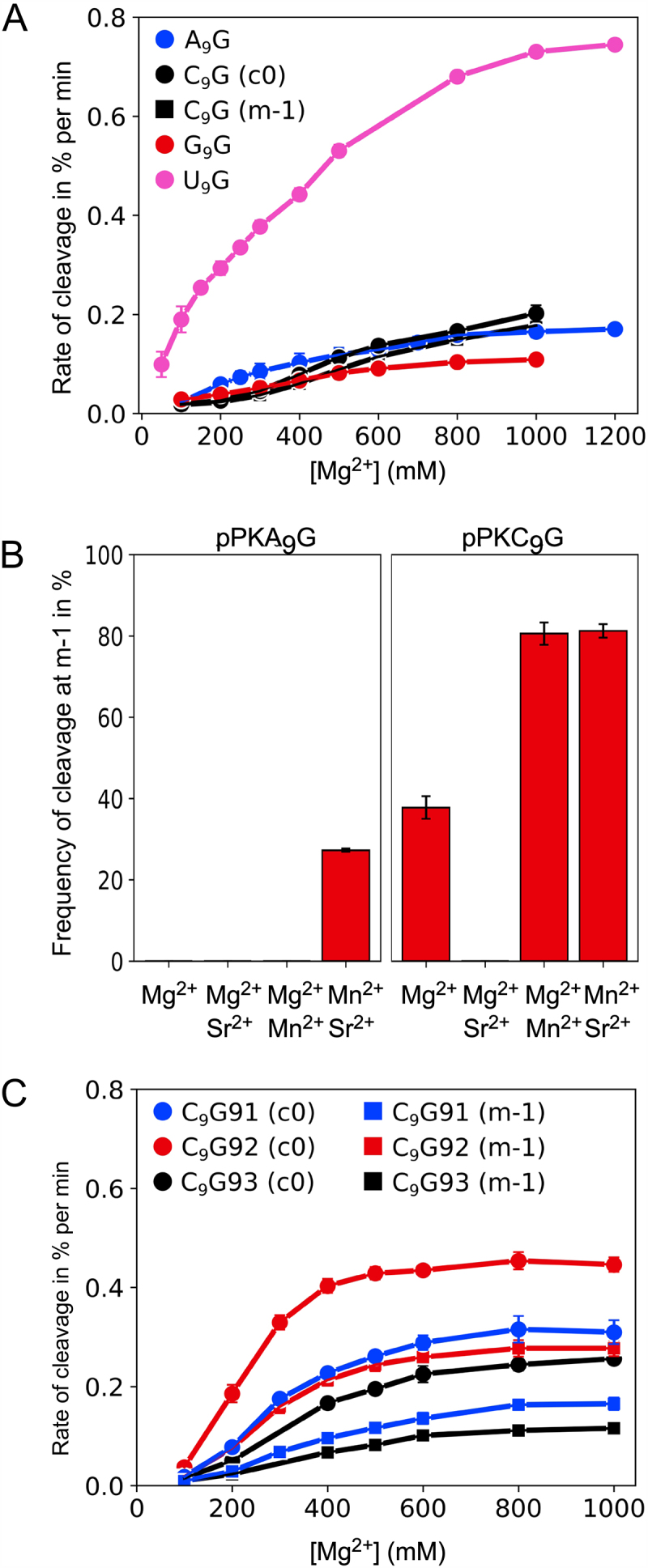
Cleavage of the pPKN_9_G variants with *Eco* RPR_wt_ as a function of Mg^2+^ concentration or different Me^2+^ combinations. (A) Mg^2+^ profiles for pPKN_9_G. The rate of cleavages as a function of Mg^2+^ concentration at c0 for pPKA_9_G_33_, pPKG_9_G_33_ and pPKU_9_G_33_ (marked as AG, GG and UG, respectively), and at c0 and m-1 for pPKC_9_G_33_ (marked as CG). The substrate concentration was ≤0.02 μM and the *Eco* RPR_wt_ concentration was 3.2 μM irrespective of substrate. The experiment was performed under single-turnover conditions at 37°C as described in Materials and Methods, and the cleavage rates (in % per min) represent the mean ± experimental errors of at least three independent experiments. (B) Frequency of cleavage of pPKA_9_G_33_ and pPKC_9_G_33_ at c0 and m-1 in the presence of Mg^2+^ (400 mM), Mg^2+^ (150 mM) and Sr^2+^ (250 mM), Mg^2+^ (250 mM) and Mn^2+^ (150 mM), and Mn^2+^ (150 mM) and Sr^2+^ (250 mM). The concentration of substrate was ≤0.02 μM while the *Eco* RPR_wt_ concentration was 6.4 μM. The experiments were performed under single-turnover conditions in buffer C (total Me^2+^ concentration 400 mM) at 37°C as outlined in Materials and Methods and time of incubation was 300 min. The frequencies at m-1 represent the mean ± experimental errors of at least three independent experiments, see also Figure legend 2C. We emphasize, for those combinations where we did not detect cleavage at m-1 are shown as zero frequency of cleavage at m-1. (C) Rates of cleavage for pPKC_9_G_33_91 (CG91) at c0 and m-1, pPKC_9_G_33_92 (CG92) at c0 and m-1, and pPKC_9_G_33_93 (CG93) at c0 and m-1 as a function of Mg^2+^ concentration. The experiment was performed under single-turnover conditions at 37°C as described in Materials and Methods, and the cleavage rates (in % per min) represent the mean ± experimental errors of at least three independent experiments (see also above under A).

*Eco* RPR_wt_ cleavage requires divalent metal ions, with a preference for Mg^2+^ considering both site selection and cleavage efficiency [70]. Hence, we first wanted to define the optimal Mg^2+^ concentration for cleavage of the pPKN_9_G_33_ variants. Determination of the cleavage rates as a function of increasing Mg^2+^ under single turnover conditions revealed that optimal rates were achieved at roughly 800 mM for the N_9_ variants with the exception of pPKC_9_G_33_. For this variant ≥1000 mM was needed (saturation was not reached) irrespective of cleavage site, c0 or m-1 (Figure 3A). Following up on this observation, we analysed cleavage in the presence of combinations of divalent metal ions. We focused on Mg^2+^/Sr^2+^, Mg^2+^/Mn^2+^ and Mn^2+^/Sr^2+^ since for model hairpin substrates addition of Mn^2+^ promotes *Eco* RPR_wt_-mediated cleavage at −1 (corresponds to m-1 in the pPKN_9_G_33_ substrates), while Sr^2+^ suppresses cleavage at this site [70,71]. In keeping with the influence of Mn^2+^ and Sr^2+^ on site selection, the C_9_ substrate was cleaved preferentially at m-1 when we added Mn^2+^ while addition of Sr^2+^ reduced cleavage at m-1 (Figure 3B; note that in these experiments the final Me^2+^ concentration was 400 mM, see Materials and methods). For the A_9_ variant, we detected increased frequency of cleavage at m-1 only in the presence of Mn^2+^ and Sr^2+^ while for the other Me^2+^ combinations (as indicated in Figure 3B), and the G_9_ and U_9_ variants (data not shown), cleavage was detected only at c0. We were unable to detect any cleavage in the presence of only Mn^2+^ or Sr^2+^ (not shown).

Together these data are consistent with a model where the N_9_ identity influences positioning of divalent metal ion(s) at and in the vicinity of the cleavage sites (see Discussion).

### The N_9_ identity affects the kinetic constant k_obs_ and U_9_ is preferred

Next, we determined the kinetic constants, k_obs_ and k_obs_/K^sto^, under single turnover conditions at saturating Mg^2+^ concentration (800 mM, non-saturating for pPKC_9_G_33_, see above and Figure 3A). The data revealed that k_obs_ for cleavage of pPKU_9_G_33_ at the c0 site was roughly six- to ten-fold higher compared to the other N_9_ variants (Table 1). Calculating K^sto^ (≈K_d_; see [23]) suggested that *Eco* RPR_wt_ binds these pseudoknot substrates with similar affinities within a factor of two and irrespective of cleavage at c0 or at m-1 (see pPKC_9_G_33_; Table 1). Substituting G at position 33 in pPKU_9_G_33_ (Figure 1A; G_33_ underlined in the GCCAC 3’ trailer, see also above) with A generated pPKU_9_A_33_. This change resulted in a modest improvement in binding and k_obs_ (cf. pPKU_9_G_33_ vs. pPKU_9_A_33_; Table 1). In conclusion, our data suggest that the identity of N_9_ primarily affects k_obs_ (with U at the N_9_ position being most favourable) while they bind to *Eco* RPR_wt_ with similar affinities (see also the Discussion).

**Table 1.** The kinetic constants k_obs_ and k_obs_/K^sto^ for cleavage of various pseudoknot substrates at 800 mM Mg^2+^ with Eco RPR_wt._

Substrate	Cleavage site	k_obs_ (min^−1^)	k_obs_/K^sto^ (min^−1^µM^−1^)	K^sto^ (≈K_d_)# (µM)
pPKA_9_G_33_	c0	(19 ± 1) x 10^−4^	(70 ± 12) x 10^−4^	0.27
pPKG_9_G_33_	c0	(12 ± 0.64) x 10^−4^	(25 ± 8.4) x 10^−4^	0.48
pPKU_9_G_33_	c0	(120 ± 11) x 10^−4^	(490 ± 140) x 10^−4^	0.24
pPKU_9_A_33_	c0	(170 ± 13) x 10^−4^	(1200 ± 290) x 10^−4^	0.14
pPKC_9_G_33_	c0	(21 ± 0.8) x 10^−4^	(70 ± 12) x 10^−4^	0.30
	m-1	(16 ± 0.72) x 10^−4^	(74 ± 3.9) x 10^−4^	0.22
pPKC_9_G_33_93	c0	(47 ± 1.3) x 10^−4^	(150 ± 14) x 10^−4^	0.31
	m-1	(21 ± 0.74) x 10^−4^	(80 ± 10) x 10^−4^	0.26

The experiments were performed under saturating single turnover conditions at 800 mM Mg^2+^ at pH 6.1 as described in Materials and Methods. The final concentration of substrate was ≤0.02 μM. The concentration of the different *Eco* RPR variants was varied between 0 and 26 μM and the concentration range varied dependent on *Eco* RPR variant and substrate. The data represent mean ± experimental errors calculated from at least three independent experiments. ^#^The K^sto^ values were calculated using k_obs_ and k_obs_/K^sto^ values.

### Factors influencing site selection in cleavage of pPK substrates

**Figure 4.**
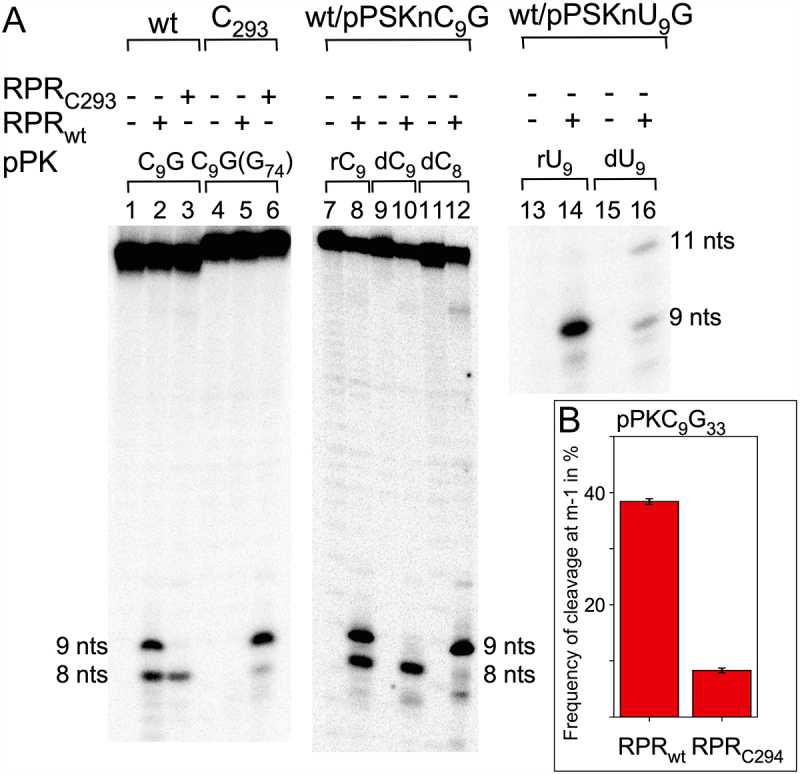
Cleavage of pPKC_9_G_33_ and pPKU_9_G_33_ variants with different *Eco* RPRs in buffer C at 800 mM Mg(OAc)_2_ as indicated. (A) Cleavage of pPKC_9_G_33_ and pPKC_9_G_33_(G_74_) with *Eco* RPR_wt_ and *Eco* RPR_C293_ and cleavage of substrates carrying dC_9_ and dU_9_ with *Eco* RPR_wt_ as indicated. The experiments were performed at 37°C in buffer C and 800 mM Mg(OAc)_2_. The substrate concentration was ≤0.02 μM, *Eco* RPR concentration 6.4 μM (lanes 2, 3, 5, 6, 8, 14 and 16) and 13 μM (lanes 10 and 12). Time of incubations: lanes 1–6, 300 min, lanes 7–12 and 15–16, 360 min, and lanes 13 and 14, 60 min. nts mark the size of products generated after cleavage at sites c0 (9 nt), m-1 (8 nt) and m+3 (11 nt). (B) Frequency of cleavage of pPKC_9_G_33_ at m-1 with *Eco* RPR_wt_ and *Eco* RPR_C294_ in the absence of C5. The substrate and RPR concentrations were ≤0.02 μM and 9.6 μM and the reactions were as described in Materials and Methods, and panel A. Time of incubation, 90 min. The frequencies represent the mean and experimental errors of at least three independent experiments, see also Figure legend 2, panel C.

The pPKA_9_G_33_ RNA lacking the 5’-GCCAC trailer (pPK) was not cleaved by *Eco* RPR_wt_ (see above). We therefore wanted to investigate the impact of the RCCA-RPR interaction (see above; interacting residues underlined) to get insight into how its structural topology affects cleavage of these pseudoknot substrates. We argued that, in accordance with our previous data, interference with this interaction would affect site selection, for a review see [72]. Hence, we substituted the C corresponding to ‘C_74_’ in tRNA with G in pPKC_9_G_33_ (the 5’-GCCAC trailer changed to 5’-GGCAC; Figure 1A). This variant, pPKC_9_G_33_(G_34_), was not cleaved by *Eco* RPR_wt_ while *Eco* RPR_C293_ (an RPR variant that restores pairing with G_74_ in cleavage of pre-tRNA, see [64]) and Figure 1D cleaved this variant mainly at c0 and with significantly higher frequency than *Eco* RPR_wt_ cleaved pPKC_9_G_33_ at c0 (Figure 4A, cf. lanes 2 and 6). By contrast, *Eco* RPR_C293_ (which cannot pair with C_34_ in pPKC_9_G_33_) cleaved pPKC_9_G_33_ almost exclusively at m-1 (see lane 3). We also analysed cleavage using *Eco* RPR_C294_ in which U_294_, that pairs with the discriminator base N_73_ in pre-tRNA (see Figure 1D; N_73_ corresponds to N_33_, see above), was replaced with C (see Figure 1A). Compared to *Eco* RPR_wt_, *Eco* RPR_C294_ cleaved pPKC_9_G_33_ with reduced frequency at the m-1 site, while the other N_9_ variants were cleaved at c0 (Figure 4B, and data not shown). Together, these findings emphasize the importance of formation and structural topology of the RCCA-RPR interaction for cleavage of pPKC_9_G_33_.

The N_−1_ 2’-OH (N_9_ in pPKN_9_G_33_) in pre-tRNA and other substrates plays a crucial role in the RPR alone reaction with respect to site selection and cleavage efficiency, see e.g. [13,25, 33–38, 73–75]. Consistent with these previous findings, a 2’-H modification at C_9_ in pPKC_9_G_33_ restricted cleavage to m-1, while a 2ʹH at C_8_ directed cleavage predominately to the c0 site (Figure 4A, cf. lanes 8, 10 and 12). Introducing a 2’-H at position 9 in the U_9_ variant also reduced cleavage at c0 but in contrast to pPKdC_9_G_33_, the dU_9_ variant was cleaved at a new site, m+3 (between residues A_11_ and G_12_ in helix 2; Figures 1A and 4A, cf. lanes 14 and 16), but not at m-1. Hence, site selection depends on both N_9_ identity and the 2’-OH at the position 5’ of the cleavage site.

Next, we asked whether structural changes at other positions in pPKC_9_G_33_ affect site selection. We argued that substitution of chemical groups of bases in the vicinity of the cleavage site would influence the charge distribution and thereby affect the positioning of Mg^2+^ near the site of cleavage (Figure 1A–C; coloured in orange). Thus, we designed and generated three pPKC_9_G_33_ variants where the orientations and identity of specific base pairs were changed (Figure 1A): pPKC_9_G_33_91 (G_12_/C_30_ changed to C_12_/G_30_), pPKC_9_G_33_92 (A_11_/U_31_ changed to G_11_/C_31_) and pPKC_9_G93 (G_12_/C_30_ changed to C_12_/G_30_ and A_11_/U_31_ changed to G_11_/C_31_). *Eco* RPR_wt_ cleaved these three variants with reduced frequency at m-1 compared to pPKC_9_G_33_ [cf. Figure 3A (C_9_G_33_) vs. 3C (variants 91, 92 and 93); the frequency of cleavage at m-1 for pPKC_9_G_33_ was 46% while for pPKC_9_G_33_91, pPKC_9_G_33_92 and pPKC_9_G_33_93 the frequencies were 31%, 38% and 34%, respectively]. Also, relative to pPKC_9_G_33_ a lower Mg^2+^ concentration was required for optimal cleavage irrespective of variant and cleavage site (cf. Figure 3A and C). Determination of k_obs_ and k_obs_/K^sto^ for pPKC_9_G_33_93 (Table 1) revealed that the reduced cleavage at m-1 is due to a ≈ two-fold increase in k_obs_ for cleavage at c0 while only a minor increase (≈30%) in k_obs_ at m-1 was detected relative to cleavage of pPKC_9_G_33_ at these sites. Noteworthy, we did not detect any substantial difference in binding, cf. K^sto^ (≈K_d_) values for pPKC_9_G_33_ and pPKC_9_G_33_93 (Table 1). Relative to the other factors discussed above, the results of changing the orientations and identity of these base pairs on cleavage were modest. Nevertheless, we interpret these data to suggest that changing the structural topology facing the cleavage site influence site selection and Mg^2+^ requirement/binding.

### Cleavage of the pPKN_9_G_33_ variants with bacterial type B and archaeal RPR

**Figure 5.**
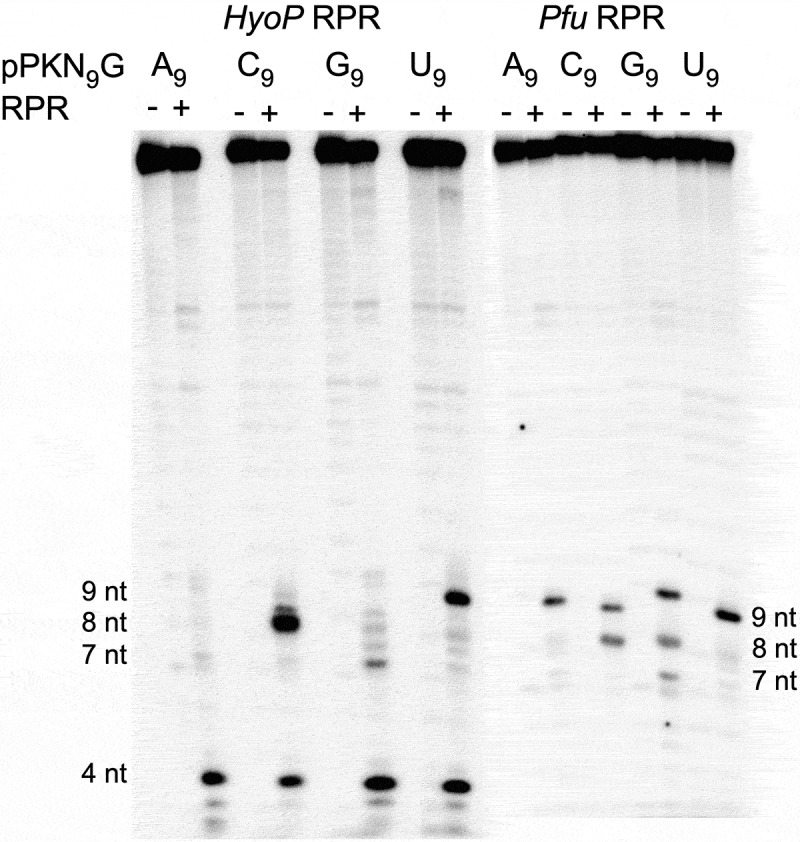
Cleavage of pPKN_9_G_33_ variants with *HyoP* RPR and *Pfu* RPR as indicated. 9, 8, 7 and 4 nt mark the size of the cleavage products as a result of cleavage at c0, m-1, m-2 and m-5 (between C_4_ and G_5_; Figure 1A). The reactions were performed without (-) and with (+) RPR for 300 min. The reactions were performed in buffer C and 800 mM Mg(OAc)_2_ at 37°C and the concentrations of substrate and RPRs were ≤0.02 μM of the indicated pPK variants (A_9_, C_9_, G_9_ and U_9_). Enzyme concentrations were 4.4 μM (*HyoP* RPR; substrates C_9_ and U_9_), 12 µM (*HyoP* RPR; substrates A_9_ and G_9_), 15 µM (*Pfu* RPR; substrates C_9_ and U_9_) and 7.4 μM (*Pfu* RPR; substrates A_9_ and G_9_).

On the basis of secondary structure bacterial RPR can be divided into type A (Ancestral) and type B (Bacillus) where *Eco* RPR represents type A. *HyoP* RPR from *Mycoplasma hyopneumoniae* is a type B representative, while the archaeal *Pyrococcus furiosus* (*Pfu*) RPR belongs to type A. Both these RPRs are catalytically active in the absence of RNase P proteins [4,52,53]. Hence, we asked whether these two RPRs can cleave the pseudoknot pPKN_9_G_33_ variants. The results in Figure 5 show that *HyoP* RPR cleaved the C_9_ variant at m-1, while the U_9_ variant was cleaved at c0. For the A_9_ and G_9_ variants, we detected very weak (if any, in particular for the A_9_ variant) cleavage mainly at m-2 between U_7_ and C_8_. Noteworthy, all the N_9_ variants were also cleaved at m-5 between C_4_ and C_5_ (Figure 1A, B). For *Pfu* RPR, we detected cleavage for all four N_9_ variants and the C_9_ and G_9_ variants were cleaved at both c0 and m-1. The G_9_ variant was also cleaved at m-2, *i.e*. between U_7_ and C_8_ (Figure 1A).

These data show that these pseudoknot RNAs also act as substrates for other RPRs but site selection varies and depends on the RPR. Conceivably this is related to structural differences. Comparing the type A *Eco* RPR and type B *HyoP* RPR, e.g. for *Eco* RPR the substrate ‘3’-RCC-motif’ interacts with an internal loop (Figure 1D) while in the case of *HyoP* RPR this interaction involves a loop structure [65]. Albeit *Pfu* RPR belongs to type A RPR, there are structural differences compared to *Eco* RPR e.g. the P18 element is lacking in *Pfu* RPR (Figure 7). Whether this is the reason why the G_9_ variant is cleaved by *Pfu* RPR at several sites (see Figure 5) requires further investigation. We also emphasize that we have reported differences in cleavage of other model substrates by *Eco* RPR vs. *Pfu* RPR [53,78].

## Modelling the interaction between Eco RPR and pPK substrates

To understand the difference in cleavage site selection for pPKC_9_G_33_ relative to the other variants we modelled the interaction between RPR and the different pPKN_9_G_33_ derivatives. For this purpose, we used the *Thermotoga maritima* RNase P (type A RPR) structure in complex with tRNA^Phe^ [50], the structure of the biotin-binding RNA aptamer [49] and the macromolecular modelling code MMB [56,61] as outlined in Materials and Methods. Traditional Molecular Dynamics (MD) is the most widely used means of dynamical atomistic physics-based modelling of macromolecules. However, MD does not work as well for RNA as it does for proteins, regarding accuracy and runtime. If an initial 3D structure is not available, MD will struggle to fold the RNA and place ions correctly in a reasonable amount of time. MacroMoleculeBuilder (MMB) can quickly convert limited information about base-pairing into 3D structure [56,61]. It can also apply Coulomb and van der Waals forces [62] and place an explicit-water droplet surrounding a region of interest. Within such droplets ions can quickly travel to form electrostatically favourable interactions with both RNA and water. MMB can quickly yield insights that help explain biochemical phenomena [61].

### Canonical and alternative cleavage events observed for the C_9_ variant

**Figure 6.**
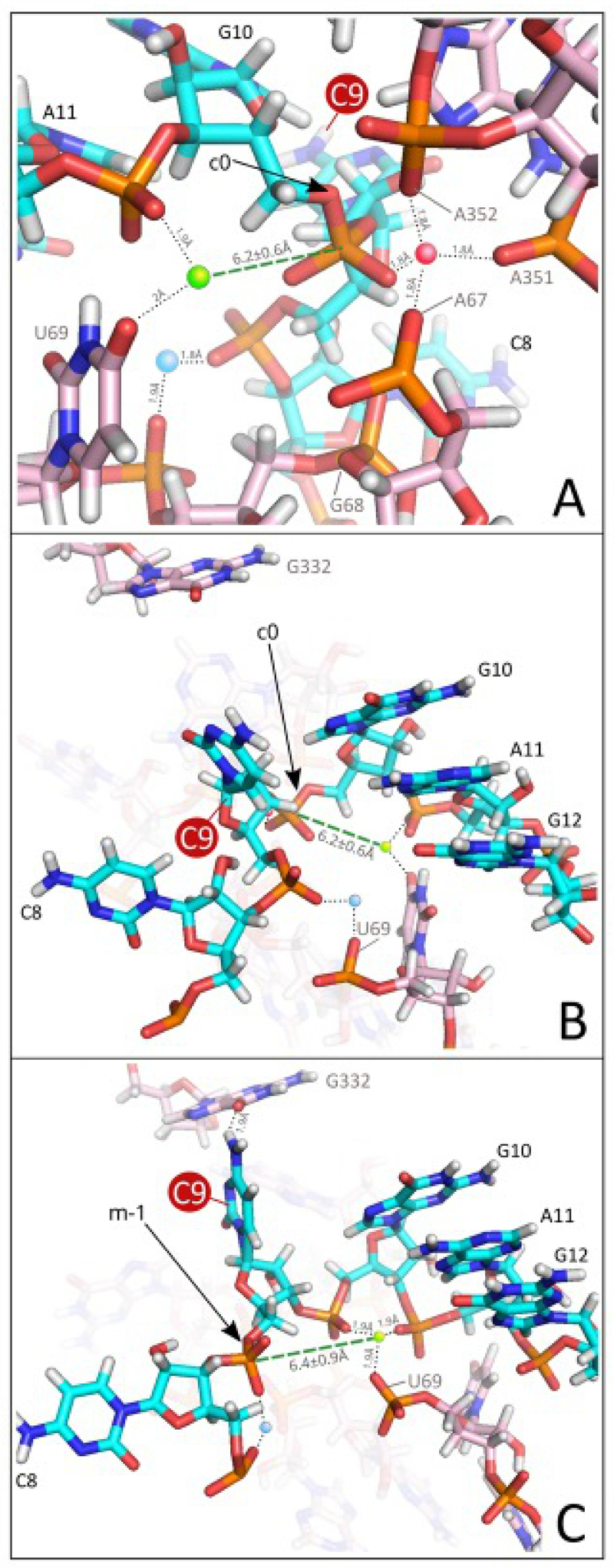
Modelling of RNase P RNA (RPR) in complex with pPKC_9_G_33_. (A) Canonical cleavage conformation displaying an inline nucleophilic attack by the Mg^2+^-activated H_2_O (green dashed line). Functional groups in the Mg^2+^ (marked in green) ligand sphere include the phosphate group linked to A_11_ (in pPKC_9_G_33_) and O-4 of U_69_ (*Eco* RPR numbering, U_69_ corresponds to U_52_ in the RNase P-tRNA structure [50]). The black arrow marks the phosphate resulting in cleavage at the canonical site c0. In this model, binding between pPKC_9_G_33_ and RPR is also mediated by a second Mg^2+^ (marked in red) surrounded by phosphate groups linked to residue G_10_ in the substrate and RPR residues A_67_, A_351_, and A_352_. (B) Alternative view of the canonical cleavage site, c0 (marked with a black arrow), and location of the Mg^2+^ (marked in green) that activates the H_2_O that acts as the nucleophile. Results shown in panels A and B are from the same simulation run. (C) Alternative cleavage conformation where the Mg^2+^ (marked in green) positioned for an in-line nucleophilic attack resulting in cleavage at m-1 (marked with a dashed green line and a black arrow, respectively) is surrounded by phosphate groups linked to G_10_, A_11_ and U_69_ (the ‘G_10_-A_11_-U_69_’ configuration; for details see main text). Pink- and cyan-coded residues represent residues in the RPR and pPKC_9_G_33_, respectively. Coloured spheres mark the position of Mg^2+^ near the cleavage sites. For clarity, other residues belonging to the complex were omitted.

**Figure 7.**
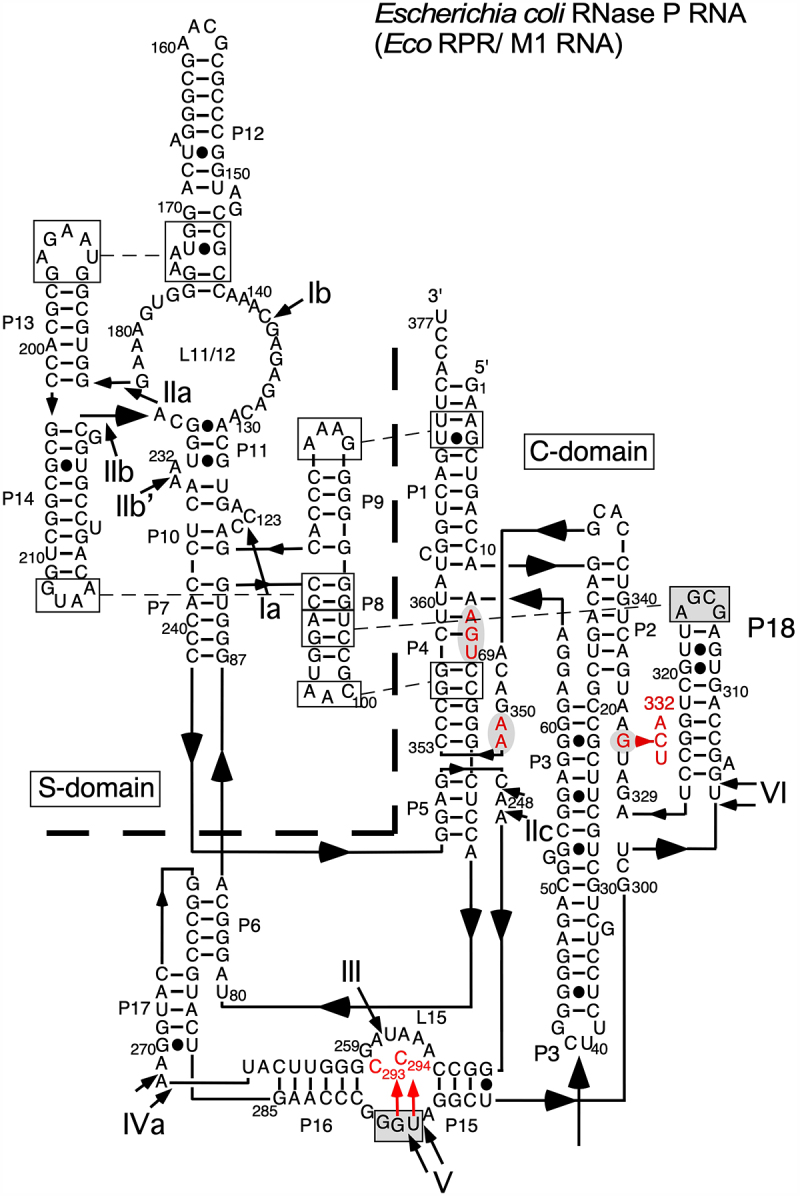
Structure model of type A *Eco* RPR according to Massire et al. [76]. Residues A_67_, G_68_, U_69_, G_332_, A_351_ and A_352_ (see main text and Figure 6) are marked in red on a grey background. The changes introduced at position 332 and in L15 are marked with red arrow (see also Figure 1C). For orientation the Pb^2+^-induced cleavage sites are included and indicated with roman numerals, see also [70,77]. The dashed line marks the demarcation between the S- and the C-domains.

Post-equilibration conformations for RNase P in complex with pPKC_9_G_33_ are shown in Figure 6. Conformations judged to induce cleavage at the c0 site (between C_9_ and G_10_; Figure 6A and B) require the presence of Mg^2+^ (marked in green), which activates H_2_O for an inline nucleophilic attack (green dashed line) from the opposite side of the scissile phosphate relative to the C_9_ 2’-OH (see e.g. Ref [39]). The distance between the Mg^2+^ and the scissile phosphate varied between 5.2 and 7.4 Å (with a mean value and standard variation equal to 6.2 ± 0.6 Å), a distance allowing space for the H_2_O responsible for the nucleophilic attack [23,39,72,79,80]. Functional groups in the ligand sphere of this Mg^2+^ include the phosphate groups of A_11_ (in pPKC_9_G_33_) and the O-4 of U_69_ (*Eco* RPR numbering and corresponding to U_52_ in the RNase P-tRNA structure [50]). Geometries compatible with an in-line canonical cleavage event (Figure 6A) were also associated with the presence of a binding configuration where an adjacent Mg^2+^ (marked in red) is surrounded by phosphate groups linked to G_10_ in pPKC_9_G_33_, and to three RPR residues A_67_, A_351_, and A_352_ (Figure 6A; *Eco* RPR numbering Figure 7; A_67_, A_351_ and A_352_ correspond to A_50_, A_313_ and A_314_ in the crystal structure of the RNase P-tRNA complex [50]; hereafter we follow *Eco* RPR numbering). Occasionally, G_68_ was also observed to be present. A second metal-ion binding site was also observed (marked in blue in Figure 6A and B), albeit its nucleotide composition was less conserved, covering any of the combinations such as C_9_-U_69_, G_10_-A_11_-G_68_, or C_9_-G_10_-A_11_-G_68_ (U_69_ and G_68_ correspond to residues in *Eco* RPR).

Presence of these two Mg^2+^ binding sites, however, did not suffice to guarantee a canonical cleavage conformation. The precise orientation of the G_10_ phosphate group depends on the conformation of the pPKC_9_G_33_ backbone in this highly populated region of the complex (Figure 6B). Backbone torsion angles are in turn affected by small variations in the position of the surrounding RPR residues, which influence the orientation of C_8_ and C_9_ in pPKC_9_G_33_. As a consequence, for several of the dynamical modelling runs the resulting equilibrium conformation did not conform to a geometry compatible with positioning Mg^2+^ for an in-line attack (see above) and cleavage at the c0 site (*i.e*. the canonical site). Instead, in a subset of these runs the position of Mg^2+^ (marked in green) favoured an in-line attack and cleavage at the alternative site (m-1) between C_8_ and C_9_ (Figure 6C, see also Figure 1). Whenever an ion was observed in position to activate the water nucleophile for cleavage at m-1, phosphate groups linked to G_10_, A_11_ and the RPR residue U_69_, referred to as the ‘G_10_-A_11_-U_69_’ configuration, always surrounded the Mg^2+^ suggested to be involved in coordinating the H_2_O nucleophile for an in-line nucleophilic attack. Our results suggest that this configuration is necessary for cleavage to occur at the alternative site m-1. Further substantiating the robustness of this model, for the set of runs involving pPKC_9_G_33_, a similar number of cleavage events were observed for the canonical and alternative configuration. These findings agree with the experimental data (see above, e.g. Figure 2B and Table 1).

### The G_12_/C_30_ to C_12_/G_30_ switch in pPKC_9_G_33_ destabilizes the configuration at the alternative cleavage site

**Figure 8.**
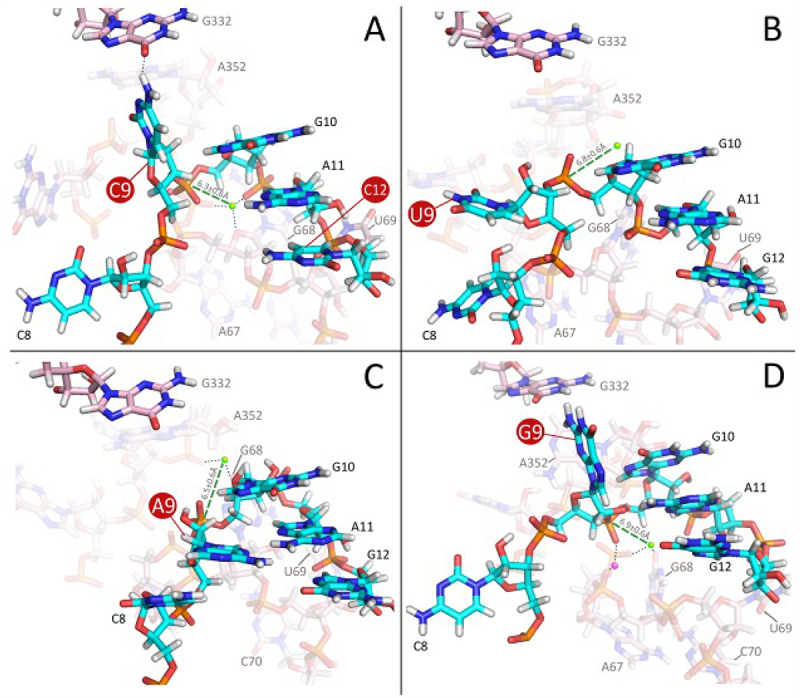
Modelling of RNase P RNA (RPR) in complex with four pPKN_9_G_33_ variants. (A) pPKC_9_G_33_91 in which G_12_/C_30_ was replaced with C_12_/G_30_. The replacement disturbs the position and binding patterns of Mg^2+^ (marked in green) positioned in the vicinity, creating a scenario where the G_10_-A_11_-U_69_-Mg^2+^ configuration required for cleavage at the alternative m-1 site is energetically less favourable. (B-D) pPKU_9_G_33_ (B), pPKA_9_G_33_ (C) and pPKG_9_G_33_ (D). The spatial orientation of the C_8_ and N_9_ side chains for the U_9_, A_9_, and G_9_ variants leads to: (I) conformation of the N_9_ phosphate group incompatible with cleavage at the alternative m-1 site and (ii) RPR-pPKN_9_G_33_ binding conformation distinct from the G_10_-A_11_-U_69_-Mg^2+^ configuration associated with cleavage at the alternative m-1 site. Pink- and cyan-coded nucleotides represent residues in the RPR and pPKN_9_G_33_, respectively. Coloured (green and pink; the Mg^2+^ generating the nucleophile are marked in green) spheres mark the positions of Mg^2+^ near the cleavage site, c0. For clarity, other residues belonging to the complex were omitted.

Introducing changes at positions distant to the cleavage sites in pPKC_9_G_33_ reduced the frequency of cleavage at m-1 (see above and cf. Figures. 1, 2C, 3A,C). Changing G_12_/C_30_ to C_12_/G_30_, as in pPKC_9_G_33_91, had minimal impact on the configuration around the canonical cleavage site c0 (Figure 8A). The number of runs yielding a conformation compatible with cleavage at the m-1 site (between C_8_ and C_9_; see Figure 1), however, decreased by 80%. This dramatic decrease was consistent with a similar change in the number of dynamical modelling runs resulting in the ‘G_10_-A_11_-U_69_’ configuration discussed above. Moreover, this configuration was retained for the few events resulting in cleavage at the alternative site m-1 confirming that this configuration is necessary for cleavage at this site. Hence, the G_12_/C_30_ to C_12_/G_30_ change seems to disturb the positioning and binding patterns of Mg^2+^ located in the vicinity, creating a scenario where the ‘G_10_-A_11_-U_69_’ configuration required for cleavage at the alternative site m-1 is energetically less favourable. This provides a rational for the change in site selection, cleavage efficiency and difference in Mg^2+^ requirement observed experimentally comparing pPKC_9_G_33_ and the pPKC_9_G_33_ ‘91–93’ variants (see above).

### For the A_9_, G_9_ and U_9_ variants the ‘G_10_-A_11_-U_69_’ configuration at the m-1 site is absent

Dynamical modelling runs for the A_9_, G_9_ and U_9_ variants suggested conformations compatible only with cleavage at the c0 site (Figure 8B–D). Depending on the N_9_ identity, substrate residues adjust their positions vis-à-vis the RPR differently and the RPR residues involved are different from those observed with the C_9_ variant (Figure 6A, B). However, this does not seem to affect cleavage at the c0 site to any significant extent since these conformations do not favour the ‘G_10_-A_11_-U_69_’ configuration associated with cleavage at the m-1 site as discussed above. In the few instances where the ‘G_10_-A_11_-U_69_’ configuration was observed, the orientation of N_9_ (N = A, G or U) is such that its phosphate group does not point in the right direction for cleavage to occur. These data are consistent with the orientation of N_9_ (N_−1_ in pre-tRNAs and model substrates) being generally dependent on base identity at this position (see [25,51] and ***Supplemental Figure S2***; S2A and S2B).

Docking the original biotin-binding RNA aptamer (pPK) into the reactive centre in the RNase P-tRNA crystal (Figure S2C) and cryo-EM *Eco* RNase P-pre(A_−2_U_−1_)-tRNA (Fig S2D) structures [50,51] by superimposing the G_10_-C_32_ pseudoknot and G_1_-C_72_ tRNA base-pairs show that pPK fit into the pocket. Also, the pre-tRNA and pPK scissile phosphates occupy overlapping positions (marked with a black arrow in Fig S2D; note, the RNase P-tRNA crystal structure represent the post-cleavage state [50]). We note that there is also space for the added 5’-GCCAC 3’ trailer needed for the establishment of the RCCA-RPR interaction and cleavage of the pseudoknot RNA (see above). One difference comparing the interaction with pre(A_−2_U_−1_)-tRNA and pPK is the stacking between residues in the pre(A_−2_U_−1_)-tRNA 5’ leader and residues in the RPR while A_9_ and C_8_ in the pseudoknot RNA do not stack on RPR residues in keeping with our modelling studies. Another is that A_9_ and C_8_ point in the opposite direction relative to U_−1_ and A_−2_ in the pre-tRNA. Finally, RPR residues positioned in the vicinity of the pPKN_9_G_33_ cleavage sites in the modelled structures discussed above are also observed in the RNase P-tRNA crystal and cryo-EM *Eco* RNase P-pre(A_−2_U_−1_)-tRNA structures (for comparison cf. Figures 6 and 8, and *Supplemental Figure S2*) [50,51].

## Impact of Eco RPR residue 332 on cleavage site selection

**Figure 9.**
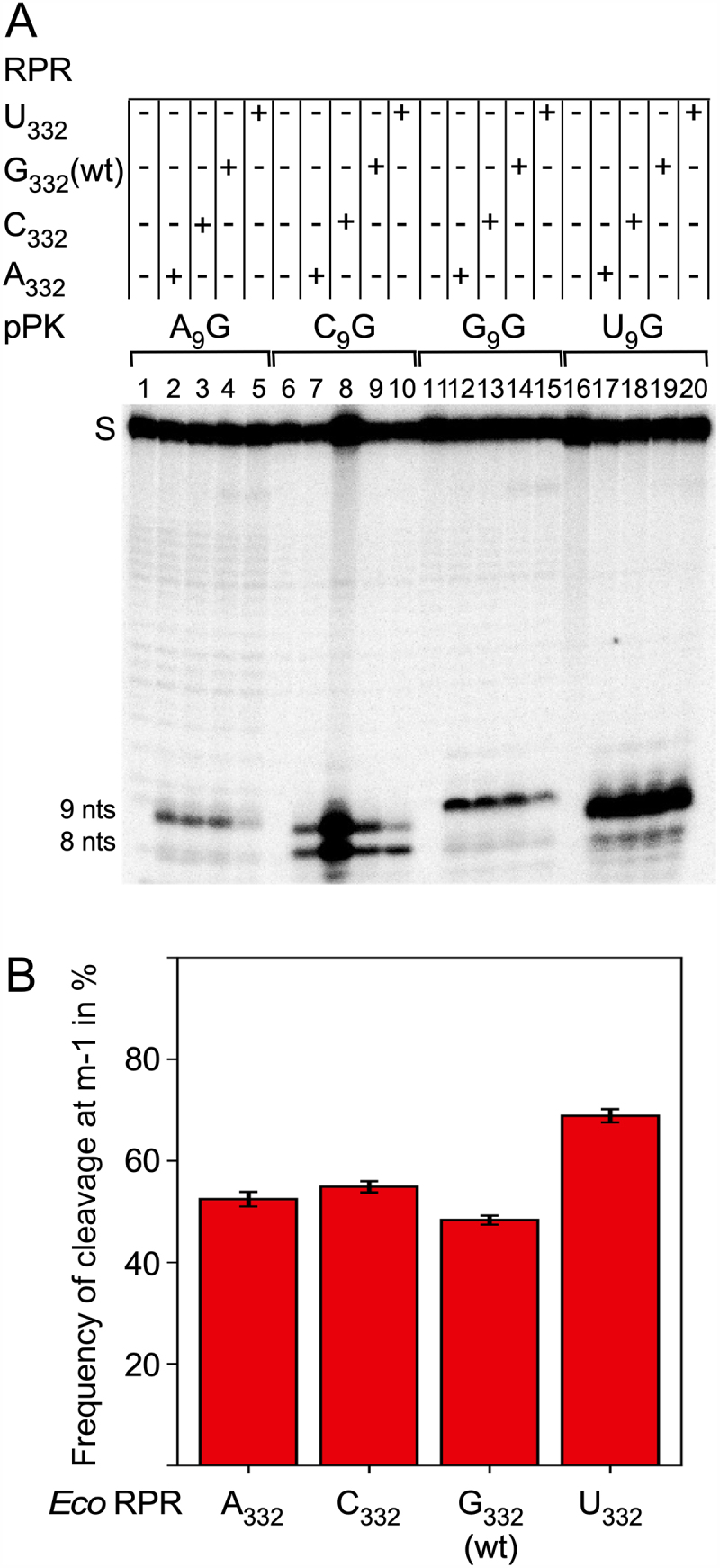
Cleavage of pPKN_9_G_33_ variants with *Eco* RPR 332 derivatives as indicated. (A) Lanes 1, 6, 11 and 16 correspond to reactions performed without RPR while the results of cleavage with the different RPR variants are presented in the other lanes as indicated. The reactions were performed in buffer C and 800 mM Mg(OAc)_2_ at 37°C as described in Materials and Methods and in Figure legends 2, 4 and 5. The concentration of substrate was ≤0.02 μM and *Eco* RPR concentrations were: lanes 7–10 and 17–20, 4.8 μM, and lanes 2–5 and 12–15, 9.6 μM. Time of incubation, 60 min. (B) Frequency of cleavage of pPKN_9_G_33_ at c0 and at m-1 with *Eco* RPR_wt_ and *Eco* RPR_C294_ in the absence of C5. The substrate and RPR concentrations were ≤0.02 μM and 4.8 or 9.6 μM and the reactions were as described in Materials and Methods. The frequencies represent the mean and experimental errors of at least three independent experiments, see also Figure legend 2B.

The modelling discussed above suggested that residue G_332_ (*Eco* RPR numbering; Figure 7) interacts with C_9_ (but not when N_9_ = A, G or U) in pPKC_9_G_33_ for cleavage at the m-1 site (Figure 6C). Hence, we substituted G_332_ in *Eco* RPR with A, C and U to further understand its role in site selection in cleavage of pPKC_9_G_33_. Changing G to U at 332 increased the frequency of pPKC_9_G_33_ cleavage at m-1 (Figure 9A, cf. lanes 10 and 7–9) while A or C mutations at position 332 in the RPR did not affect site selection to any significant extent relative to wild-type *Eco* RPR with G_332_ (Figure 9A and B). Determinations of the rate constant, k_app_, revealed that the increased cleavage at m-1 is attributable to an almost four-fold reduction in k_app_ for cleavage at the c0 site, while only a marginal effect was detected for cleavage at m-1 (cf. G_332_ vs. U_332_; Table 2). The modelling suggested that the guanosine (G_332_) carbonyl oxygen, O-6, possibly forms an H-bond with the exocyclic amine of C_9_ during m-1 cleavage (Figure 6C). Given that U_332_ carries two carbonyl oxygens at positions 2 and 4 provides one possible reason for the increased frequency of cleavage at m-1 by enabling H-bonding and ‘trapping’ C_9_ thereby favouring the ‘G_10_-A_11_-U_69_’ configuration. This is supported by the data where we modelled the U_332_ variant in complex with the pPKC_9_G_33_ substrate (*Supplemental Figure S3A*) and simulations indicating increased cleavage at the m-1 site for the U_332_ compared to the other variants (*Supplemental Figure S3B*).

**Table 2.** The rate constant k_app_ for cleavage of pPKC_9_G_33_ with *Eco* RPR 332 variants at 800 mM Mg^2+.^

Substrate	Cleavage site	k_app_ (min^−1^ pmol^−1^)
*Eco* RPR_G332_(wt)	c0	(57 ± 7.7) x 10^−3^
	m-1	(49 ± 7.6) x 10^−3^
*Eco* RPR_A332_	c0	(42 ± 5.3) x 10^−3^
	m-1	(46 ± 6.1) x 10^−3^
*Eco* RPR_C332_	c0	(30 ± 2.6) x 10^−3^
	m-1	(36 ± 0.31) x 10^−3^
*Eco* RPR_U332_	c0	(16 ± 1.7) x 10^−3^
	m-1	(34 ± 0.27) x 10^−3^

The experiments were performed under saturating single turnover conditions at 800 mM Mg^2+^ at pH 6.1 as described in Materials and Methods. The concentrations of *Eco* RPR and substrate were 0.8 and ≤0.02 µM, respectively. The data represent mean ± experimental errors calculated from at least three independent experiments.

## Discussion

RNase P cleaves several different types of RNA substrates, albeit involvement in the processing of pre-tRNAs is considered to be its main function in the cell. Here, we showed that a small RNA aptamer acts as a substrate for *Eco* RPR with and without the RNase P protein, C5, as well as for the type B *HyoP* RPR and the archaeal type A *Pfu* RPR. The RNA aptamer folds into an H-type pseudoknot structure and was selected for binding biotin [49,81; see also 48,82]. That RNase P cleaves RNA pseudoknots agrees with previously reported data [26–30]. However, the present study extends our understanding of the factors that contribute to cleavage of pseudoknot substrates, such as the importance of the N_9_ identity, the RCCA-RPR interaction, and charge distribution and Mg^2+^ binding near the cleavage site. Together, our findings provide new insight into the structural architecture and positioning of Mg^2+^ at the cleavage site that influence the choice of cleavage site [see also 25].

### The structural architecture at and in the vicinity of the cleavage site varies dependent on substrate

For *Eco* RPR, a C at N_9_ resulted in cleavage at two positions, c0 and m-1, with almost equal frequencies, while the substrates with A, G or U at this position were cleaved preferentially at one site, c0 (between N_9_ and G_10_; Figure 1A). Our modelling studies suggested that cleavage at the alternative position m-1 depends on a configuration referred to as ‘G_10_-A_11_-U_69_’, which is not favoured in pseudoknots with A, G or U at N_9_. Residue G_332_ (Figure 7) is predicted to stabilize this configuration through hydrogen bonding between the G_332_ carbonyl oxygen O-6 and the C_9_ exocyclic amine (2-NH_2_-group; Figure 6C). Accordingly, our data revealed that replacing G_332_ with U resulted in cleavage mainly at m-1 (the alternative cleavage site) supporting the importance of residue 332 with respect to the structural architecture at and in the vicinity of the cleavage site. It is conceivable that U_332_ stabilizes the ‘G_10_-A_11_-U_69_’ configuration through hydrogen bonding between the U_332_ carbonyl oxygen O-4 and the 4-amino group of C at the N_9_ position more efficiently than G_332_. This would provide a rationale for the observed increase in cleavage at the alternative site m-1 with *Eco* RPR_U332_. In this context, we note that cross-linking data position G_332_ in close proximity of the 5’ terminal G in tRNA [83] and the U_−1_ (U_−1_/N_−1_ corresponds to N_9_ in pPKN_9_G_33_) in a model pre-tRNA [84,85]. Also, the positioning of G_332_ in bacterial RNase P-pre-tRNA cryo-EM structures depends on the identities of N_−2_ and N_−1_ in the pre-tRNA 5’ leader (Figure S2A, B) [51]. Together, this raises the possibility that G_332_ interacts similarly with other substrates carrying a C immediately 5’ of canonical cleavage sites as observed in the pseudoknot substrates. For example, in mycobacteria, pre-tRNAs with C at the N_−1_ position are abundant [86], which might have an impact on the RNase P processing step. However, whether this affect cleavage site selection of native pre-tRNAs warrants for further studies.

The universally conserved bulged U_69_ (*Eco* RPR numbering; U_52_ in the RNase-tRNA structural complex; Figure 7) [50]; is also positioned close to pPKN_9_G_33_ cleavage sites and the Mg^2+^ predicted to generate the water nucleophile in our modelling. The importance of U_69_ is in keeping with previous reports [50, 87–99]. Moreover, the mechanism of RNase P-mediated cleavage is evolutionary conserved [40,50, 100–102] and in the recent cryo-EM structure of yeast RNase P in complex with a pre-tRNA A_91_, U_92_ and U_93_ (yeast numbering) are positioned near the scissile phosphate, where U_92_ corresponds to U_69_ in *Eco* RPR [100]. In the bacterial RNase P-tRNA and RNase P-pre-tRNA structures U_69_ is also positioned near the canonical cleavage site [51,98]. Together this lends support to the validity of our modelling. Previous data further suggested that in cleavage of pre-tRNA by bacterial RPR, the U_69_ carbonyl oxygen O-4 coordinates Mg^2+^, and modelling suggested that this Mg^2+^ activates the water that acts as the nucleophile [99, and references therein]. This is also one outcome of our modelling studies of cleavage of pPKC_9_G_33_ at the c0 site (Figure 6B) while for cleavage at m-1 our modelling data suggest that the *Rp*O of U_69_ fulfils this role (Figure 6C).

Earlier, we reported the influence of the identity of residue N_−1_ on site selection and the kinetics of cleavage using a model hairpin substrate, pMini3bp (Figure 1E). The pMini3bp substrates used in these studies [23,25] and the pPKN_9_G_33_ variants are of similar size, and they cannot interact with the RPR region that binds the ‘T-stem-loop’ of pre-tRNAs [22,50,103]. Albeit both these substrates are cleaved preferentially at the junction of double and single-stranded regions, the structural organization of their respective 5’ leaders differ. Comparing the impact of the N_−1_/N_9_ identity on cleavage reveals striking differences (here we only compare the substrates having A_−1(9)_G, C_−1(9)_G, G_−1(9)_G, U_−1(9)_G and U_−1(9)_A; cf. Figure 1A, E). The pPKN_9_G_33_ substrates bind with 10-fold higher affinities to *Eco* RPR_wt_ compared to the corresponding pMini3bp variants with ‘G_−1(9)_G’ being the poorest binder for both substrate types. The improved binding might be attributed to that the structure of the pPKN_9_G_33_ ‘5’-leader’, with its two unpaired residues, displays a more defined and pre-organized structure compared to the more flexible pMini3bpN_−1_G 5’ leader (Figure 1A, E); cf. K_d_ values in Table 1 vs. Table 1 in Ref [23] and might be related to that the entropic penalty for binding to the RPR is reduced for the pPKN_9_G_33._

**Scheme 1:**
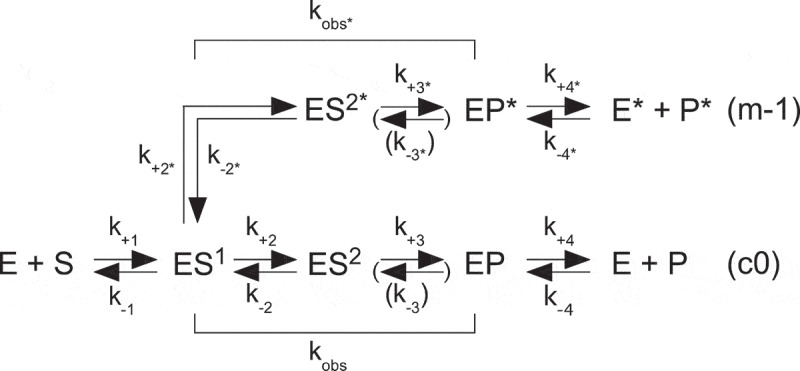

According to the simplified reaction scheme 1, following initial binding ES^1^ undergoes a conformational change prior to cleavage [see e.g. Refs 23,78; see also Ref 104 and references therein]. The pMini3bp substrates are cleaved with higher efficiencies than the pPKN_9_G_33_ variants. The k_obs_ values for pMini3bpN_−1_G vary between 4.2 min^−1^ (‘U_−1_G’) and 0.0088 min^−1^ (‘G_−1_G’), which is almost 500-fold lower than k_obs_ for the ‘U_−1_G’ variant (Table S1 and Ref 23). For the pPKN_9_G_33_ variants, we detected approximately a 10-fold difference in k_obs_ with the highest values for the variants having U at N_9_ (Table 1 and S1; pPKU_9_G_33_ and pPKU_9_A_33_; noteworthy, U is also present 5’ of the RNase P cleavage site in the TYMV tRNA-like structure, another pseudoknot substrate) [30]. Hence, the difference in k_obs_ for the pPKN_9_G_33_ variants relative to the pMini3bpN_−1_G vary between 350- (U_−1_ vs. U_9_) and 7-fold (G_−1_ vs. G_9_). Using the k_obs_ values and ΔΔG = -RTln(k_obs_)pPKN_9_G_33_/k_obs_(pMini3bpN_−1_G) [105] this amounts to a difference of 2.2 to 3.6 kcal (comparing the A_−1_/A_9_, U_−1_/U_9_ and C_−1_/C_9_ variants) and 1.2 kcal comparing the G_−1_/G_9_ variants (Table S1) in stabilization of the transition states. This would be in keeping with higher activation barriers for the pPKN_9_G_33_ relative to the pMini3bpN_−1_G variants that might be linked to the more defined and pre-organized structures of the ‘5’-leaders’ of the pPKN_9_G_33_ variants (see also Figure S2). Also, the identity of the pMini3bp N_−1_ residue has a larger impact on transition state stabilization than in cleavage of the pPKN_9_G_33_ variants (larger variation in ΔΔG values for pMini3bp relative to pPKN_9_G_33_; Table S1). In this context, cleavage of both pre-tRNA and model hairpin substrates show that structured 5’ leaders and base-pairing between residues N_−2_ (and/or N_−1_) and the first C residue in the CCA-motif and/or the discriminator base influence cleavage rates [25, 106–108],].

From our data, it is also apparent that addition of the C5 protein did not affect site selection except in the case of pPKC_9_G_33_ where we detected a small decrease of cleavage at m-1 relative to cleavage without C5 (Figure 2C). This is in contrast to cleavage of, for example, pMini3bpG_−1_G, which is cleaved ≈40% at m-1 without C5 and almost exclusively at c0 with C5 (cf. Figure 2E and F) in [23]. Addition of C5 also suppresses cleavage of pre-tRNA^Ser^Su1 at m-1 (*i.e*. −1) with *Eco* RPR variants that cannot establish a productive RCCA-RPR interaction [108]. (Noteworthy, the wt pre-tRNA^Ser^Su1 carries a C at N_−1_; for impact of C5 on site selection using other pre-tRNAs see also e.g. [35,107,109]). The C5 protein interacts with N_−8_ – N_−3_ in the ‘single-stranded’ 5’ leaders [63,110,111]. Given that N_−7_ – N_−3_ are part of helix 1 in the pPKN_9_G_33_ substrates, it is unlikely that C5 binds to these residues (see e.g. Figure 1 and S2D) as observed for N_−1_ and N_−2_ in the *Eco* RNase P-pre-tRNA cryoEM structures [51]. We also emphasize that pMini3bpG_−1_G was cleaved both at c0 and m-1 (+1 and −1, respectively) with almost equal frequencies (cf. Figure 2) in [23], while pMini3bpC_−1_G was cleaved mainly at c0 (+1). This is opposite to what we found using pPKG_9_G_33_ and pPKC_9_G_33_, which were cleaved mainly at c0 (pPKG_9_G_33_) and both at c0 and m-1 (pPKC_9_G_33_) (Figure 2B). Taken together, these differences might be related to the distinct structural organization of the 5’ leader and the architecture of the two types of substrates, which changes the role of the leader in substrate docking and formation of active site architecture (see also Figure S2D).

The RCCA-RPR interaction plays an important role for site selection in cleavage of pre-tRNA and model substrates [64,65,112]. The R residue corresponds to the discriminator base at position 73 in tRNA (see above), and it pairs with U_294_ in the *Eco* RPR-substrate complex and is referred to as the +73/294-interaction (Figure 1D [50,71]). Site selection in cleavage of the pseudoknot substrate pPKN_9_G_33_ (in particular pPKC_9_G_33_) also depends on the structural topography of the RCCA-RPR and +73/294-interactions (this study). This is consistent with previous findings, where different pre-tRNAs and model substrates were used [37,71,112]. In this context, we note that based on studies using variants of plant tRNA-like structures it was proposed that residues at −1 and +73 are important for efficient cleavage [30]. These authors also observed cleavage of pseudoknot substrates having a pyrimidine or purine at the position corresponding to N_−1(9)_ but a deeper analysis of this finding was not done. Together, this suggests that the structural topography of the distant RCCA-RPR interaction has an impact on site selection and catalysis irrespective of substrate. In this context, the N_9_ residue is most likely not in position to pair with residue G_33_ that corresponds to the tRNA discriminator base in pPKN_9_G_33_ (Figure 1A-C). However, we cannot entirely exclude pairing between C_9_ and G_33_. Nevertheless, the RCCA-RPR interaction anchors the substrate: conceivably it affects the structure near the cleavage site and thereby influencing the positioning of catalytic Mg^2+^-ion(s) (see also [37,64,71,112]).

In summary, our data support a model where the structural architecture at, and in the vicinity of, the cleavage site varies dependent on substrate with N_−1(9)_ having a key role. As a consequence, and in addition to the impact of the RCCA-RPR interaction, this would influence the positioning of Mg^2+^ involved in generating the water nucleophile and/or stabilization of the developing oxyanion in the transition state. In this context, we previously reported that structurally different cleavage sites are aligned differently in the *Eco* RPR ‘active site’ [84,85; see also 25].

### The structural architecture at, and in the vicinity of, the cleavage site has an impact on positioning of Mg^2+^important for catalysis

RPR-mediated cleavage depends on divalent metal ions and considering both efficiency and site selection, Mg^2+^ is preferred ([70]; for a review see [39]). In the bacterial RNase P-tRNA and RNase P-pre-tRNA complexes, two Me^2+^-ions are positioned near the tRNA 5’ terminal phosphate [50,51,113,114]. Hence, it is likely that these are involved in generating the water nucleophile and transition state stabilization [98]. Two Mg^2+^ are also positioned close to ‘A_9_G_10_’ in pPK (without the 5’-GCCAC trailer; Figure 1B, C) and one in the vicinity of the corresponding residues in the TYMV tRNA-like structure [47,49]. In keeping with this, our unpublished data show that both Pb^2+^ and Mg^2+^ induce cleavage at positions near N_9_ in the pPKN_9_G_33_ variants consistent with the presence of Mg^2+^ at this location (not shown). Hence, in the case of pPKA_9_G_33_, one or both Mg^2+^ are positioned such that they might take part in catalysis (see also our modelling data above). In this context, we note that based on Me^2+^-induced cleavage data with both pre-tRNA and model hairpin substrates, it was suggested that a substrate coordinating a Mg^2+^ near the cleavage site is the true substrate for RNase P [73,115,116].

Cleavage of pPKC_9_G_33_ required a higher concentration of Mg^2+^ for optimal cleavage compared to the other N_9_ variants. By changing the orientation of base pairs in helix 2 (see pPKC_9_G_33_ ‘91–93’ variants) alter the chemical groups of the bases facing C_9_ and hence would affect the charge distribution in the vicinity of the cleavage site (Figure 1B, C). These changes resulted in lower Mg^2+^ requirement to reach optimal cleavage rates and increased the frequency of cleavage at the c0 site. Also, this resulted in an 80% reduction in the number of dynamical modelling runs compatible with the ‘G_10_-A_11_-U_69_’ configuration (note that the Mg^2+^ generating the nucleophile in this configuration is coordinated differently, U_69_ *Rp*O vs. O-4, compared to cleavage at c0; Figure 6). Our data also showed that the cleavage site shifts in response to the addition of Mn^2+^ and Sr^2+^, which is in keeping with previous data [34,70,71]. Together, these data are consistent with a model where one (or both) of the Mg^2+^ is positioned near ‘A_9_G_10_’ in pPK participate in catalysis ([39,50]; Figure 1B, C). Moreover, altering the structural architecture, such that the aforementioned charge distribution is changed, influences positioning of this (or these) Mg^2+^ ions. In this context, we emphasize that substituting residues in the T-loop of yeast tRNA^Phe^ affects the positioning of Pb^2+^ (such that Pb^2+^ hydrolysis in the D-loop is affected) [79, 117–119].

As for other substrates, the 2’-OH immediately 5’ of the cleavage sites plays an important role for cleavage at this position. For pPKC_9_G_33_, introducing a 2’-H at C_9_ shifted cleavage to the alternative site m-1 while having a 2’-H at C_8_ resulted in cleavage preferentially at c0. This is in contrast to what would be predicted on the basis of the 2’-OH model, which states that the RPR binds the 2’-OH at N_−1_ in pre-tRNAs and influences cleavage at other positions [120,121]. However, the influence of changing the 2’-OH to 2’-H at N_9_ depends on the base identity since the pPKdU_9_G_33_ variant did not abolish cleavage at c0 (but it was significantly reduced) and cleavage was detected at a new site, the m+3 site in helix 2, but notably not at m-1 (Figure 4; see also [25,75] for discussion). On the basis of this finding, it is unlikely that changing the U_9_ 2’-OH to 2’-H promotes the formation of the ‘G_10_-A_11_-U_69_’ configuration, which according to our modelling is associated with cleavage at the m-1 site (see above). We and others have argued that the 2’-OH at N_−1_ acts as an inner or outer sphere for Mg^2+^ binding at the cleavage site, thereby affecting substrate binding and catalysis ([34,37] but see [36] for an alternative model). Thus, changing the 2’-OH to 2’-H would likely change the structural architecture at the cleavage site, resulting in a shift in the positioning of Mg^2+^, which leads to cleavage at alternative sites.

### Concluding remarks

In many plant RNA viruses, the tRNA-like structure at the 3’ termini is amino-acylated. For the TYMV virus, valylation is required for its amplification and enhancement of gene expression. It has been discussed that the valylation of the 3’ termini has a role in regulation of gene expression vs. replication of the positive strand, for a review see [122]. *In vitro* studies show that RNase P cleaves TYMV RNA and generate a fragment that includes the 3’ terminus [28–30]. Hence, this raises the possibility that this cleavage event is one step in this regulatory circuit by affecting enhancement of gene expression and amplification. In this context, it might be possible to adapt the EGS or the M1GS technology [123 and references therein] to develop ribozymes that cleave the TYMV 3’ end (or the 3’ end of other RNA plant virus) efficiently and use these to influence/prevent virus amplification and gene expression *in vivo*.

Apart from having a possible role in releasing the 3’ terminal tRNA-like structure from certain RNA viral genomes, pseudoknots present in mRNAs might be potential targets for RNase P as well. RNase P has been reported to process/influence processing of several mRNAs [11,14,20,24 and unpublished data] and whether the cleavage sites in these mRNAs is linked to pseudoknots is not known but one mRNA, the T2 and T4 bacteriophage gene 32 mRNA, is of particular interest. This mRNA is claimed to be a substrate for RNase P [30] and it contains a pseudoknot structure of the H-type followed by a 3’ ACCA sequence [42–44]. Whether RNase P recognizes this pseudoknot (and other pseudoknot structures embedded in mRNAs) and cleaves it in accordance with our present data, and those reported by others, warrants further studies. Here, binding of biotin to pseudoknots might be one tool to use in the process of identifying pseudoknot structures.

Finally, we note that cleavage by PRORP1 (proteinous RNase P-like activity) is affected by the N_−1_ identity with C_−1_ resulting in cleavage at −1 [124–126]. Given that the structures of substrates near the cleavage site vary, this implies that the structural architecture of the PRORP active sites change in a substrate-dependent manner and that N_−1_ has a key role. It would therefore be interesting to understand whether specific active site configurations influence the choice of cleavage site also in PRORP-mediated cleavage.

## Supplementary Material

SUPPLEMENTAL KOSEK ET AL R2.pdf

## Data Availability

The MMB executable is available as a docker container, which can be pulled as samuelflores/mmb-ubuntu, while the code is available at https://github.com/samuelflores/MMB. The input files created for this work are available at https://github.com/LLN273/RNaseP_pseudoknot. All data and materials are available from the corresponding author.
