# Supplementary material for: RNase P cleavage of pseudoknot substrates reveals differences in active site architecture that depend on residue N-1 in the 5’ leader
Source: RNA Biol. 2025 Jan 20;22(1):1–19. doi: 10.1080/15476286.2024.2427906 (PMC12710915; doi:10.1080/15476286.2024.2427906)
Supplement: SUPPLEMENTAL KOSEK ET AL R2.pdf [file KRNB_A_2427906_SM6989.pdf]

RNase P cleavage of pseudo-knot substrates reveals differences in active site architecture that depend on residue N_{-1} in the 5' leader

David Kosek[2], J. Luis Leal[3], Ema Kikovska-Stojanovska[4], Guanzhong Mao[1], Shiying Wu[5], Samuel C. Flores[6] and Leif A. Kirsebom[1]*

Supplemental information

Table S1, Figures S1, S2 and S3

Table S1. ΔΔG values comparing cleavage of $pPKN_9G_{33}$ vs pMini3bpNG at 800 mM Mg^{2+} with *Eco* RPR_{wt}

Substrate	Cleavage site	k_{obs} (min^{-1})	$\Delta\Delta G^1$ (kcal)	$\Delta\Delta G^2$ (kcal)	$\Delta\Delta G^3$ (kcal)
$pPKA_9G_{33}$	c0	19×10^{-4}	1.1		
				2.5	
$pMini3bpA_{-1}G$	c0(+1)	1100×10^{-4}			2.2
$pPKG_9G_{33}$	c0	12×10^{-4}	1.4		
				1.2	
$pMini3bpG_{-1}G$	c0(+1)	88×10^{-4}			3.8
$pPKU_9G_{33}$	c0	120×10^{-4}	$R^{\#\#}$		
				3.6	
$pMini3bpU_{-1}G$	c0(+1)	42000×10^{-4}			$R^{\#\#\#}$
$pPKU_9A_{33}^{\#}$	c0	170×10^{-4}	-0.21		
				3.5	
$pMini3bpU_{-1}A^{\#}$	c0(+1)	48000×10^{-4}			-0.08
$pPKC_9G_{33}$	c0	21×10^{-4}	1.1		
				2.2	
$pMini3bpC_{-1}G$	c0(+1)	6000×10^{-4}			1.2

Change in $\Delta\Delta G^{\#}$ values were calculated using k_{obs} values for $pPKN_9G_{33}$ (Table 1) and for pMini3bpNG (taken from Table 1 in Ref [23]), and $\Delta\Delta G = -RT\ln k_{obs}/k_{obs(reference)}$ [103].

$^{\#}$Mark the substitutions of G_{33} to A_{33} in $pPKU_9A_{33}$ and $pMini3bpU_{-1}A$.

$^1\Delta\Delta G = -RT\ln(k_{obs})pPKN_9G_{33}/(k_{obs})pPKU_9G_{33}$ (using $pPKU_9G_{33}$ as reference; $R^{\#\#}$).

$^2\Delta\Delta G = -RT\ln(k_{obs})pPKN_9G_{33}/(k_{obs})pMini3bpN_{-1}G$.

$^3\Delta\Delta G = -RT\ln(k_{obs})\ pMini3bpN_{-1}G/(k_{obs})pPKU_9G_{33}$ (using $pMini3bpU_{-1}G$ as reference; $R^{\#\#\#}$).

Figure S1

Mapping of pPSKnN$_9$G cleavage sites with nuclease P1.

Incubation without *Eco* RPR$_{wt}$ or nuclease P1: lane 1 (pPSKnA$_9$G$_{33}$), lane 4 (pPSKnC$_9$G$_{33}$), lane 7 (pPSKnC$_9$G$_{33}$) and lane 10 (pPSKnU$_9$G$_{33}$).

Incubation with nuclease P1: lane 2 (pPSKnA$_9$G$_{33}$), lane 5 (pPSKnC$_9$G$_{33}$), lane 8 (pPSKnC$_9$G$_{33}$) and lane 11 (pPSKnU$_9$G$_{33}$).

Incubation with *Eco* RPR$_{wt}$: lane 3 (pPSKnA$_9$G$_{33}$), lane 6 (pPSKnC$_9$G$_{33}$), lane 9 (pPSKnC$_9$G$_{33}$) and lane 12 (pPSKnU$_9$G$_{33}$).

Negative controls and reactions with *Eco* RPR$_{wt}$ were performed at 37°C with 800 mM $Mg(OAc)_2$ in buffer C [50 mM MES (final pH 6.1), 0.8 M NH_4OAc]. The incubation time was 90 min and *Eco* RPR$_{wt}$ concentrations of were 6.4 µM (pPSKnC$_9$G$_{33}$, pPSKnU$_9$G$_{33}$) and 13 µM (pPSKnA$_9$G$_{33}$, pPSKnG$_9$G$_{33}$). Reactions with nuclease P1 were performed in buffer P1 (40 mM NaOAc, pH 5.3, 0.5 mM $ZnSO_4$). The RNA substrate was denatured at 95ºC for 5 min and then added to prewarmed (70ºC) buffer P1 followed immediately by the addition of one ng (corresponding to ≥0.0002 units) nuclease P1 and incubated for one min.

Fig S1

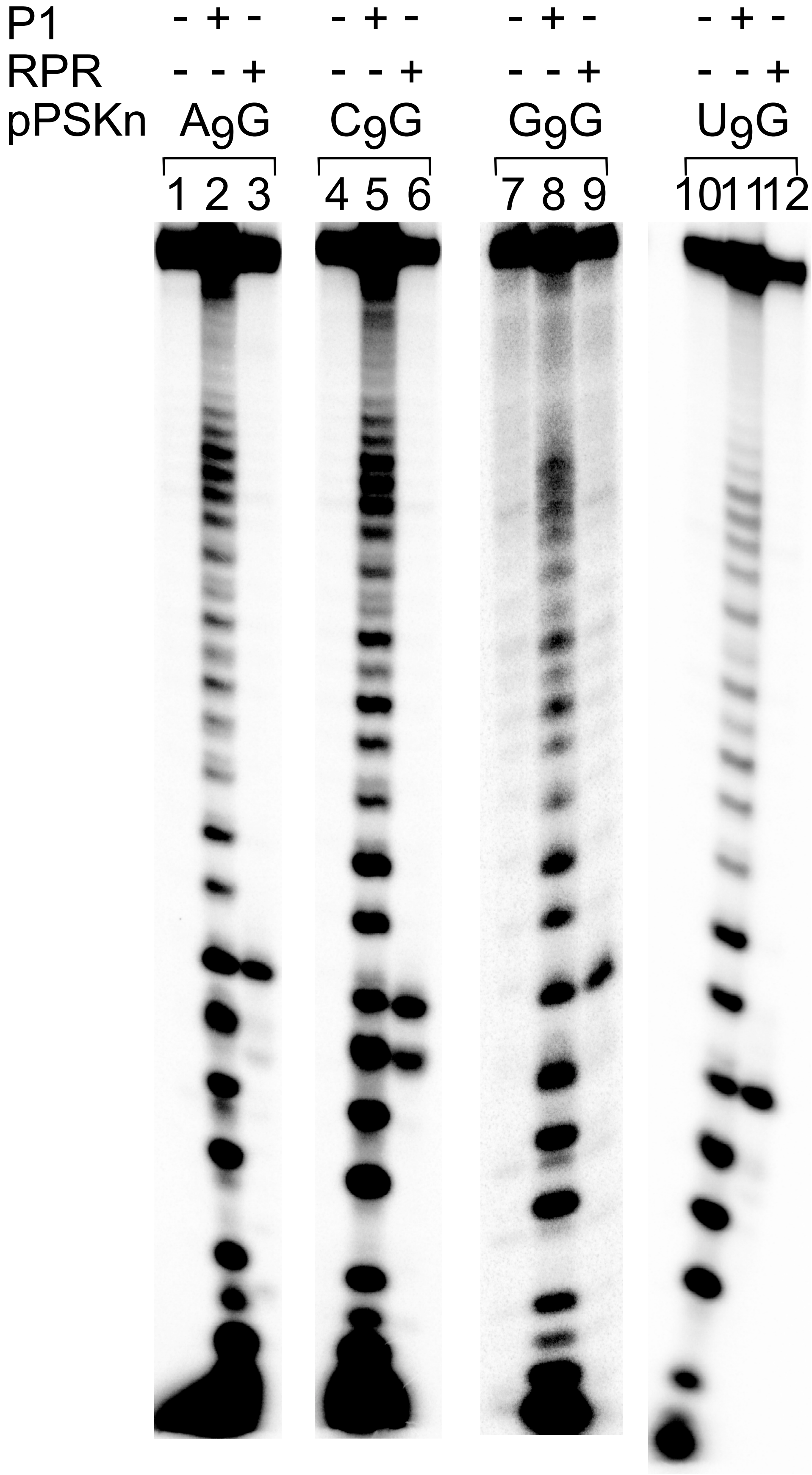

Figure S2

(A) and (B) Position of RPR residues in cryo-EM *Eco* RNase P-pre-tRNA complexes. Pre-tRNA residues are colored in orange, in (A) numbered A_{-2}, U_{-1}, G_1, G_2 and C_3, and in (B) G_{-2}, G_{-1}, G_1, G_2 and C_3. In (A) and (B) the "green-blue" color marks the *Eco* RPR residues (see Figure 7). The black arrows mark the RNase P cleavage site. The images were created with PyMOL (Schrödinger, LLC), and PDB 7UO0 and PDB 7UO1 [51].

(C) and (D) Rigid-body docking of the original biotin RNA aptamer structure pPK (PDB 1F27) into the RNase P reactive center using the Coot version 0.9.8.95.

(C) The pPK G_{10}-C_{32} base pair superimposed on the tRNA G_1-C_{72} base pair in the RNase P-tRNA crystal structure (PDB 3Q1R) representing the post-cleavage state.

(D) The pPK G_{10}-C_{32} base pair superimposed on the tRNA G_1-C_{72} base pair in the RNase P-pre($A_{-2}U_{-1}$)-tRNA cryoEM structure (PDB 7UO1) representing the pre-cleavage state.

In (C) and (D) RPR residues are colored in purple, pPK residues in blue, and tRNA (C) and pre-tRNA (D) residues in yellow. Relevant residues are indicated. The pPKN_9G_{33} and pre-tRNA scissile phosphates are marked with black arrows. The images were created with PyMOL (Schrödinger, LLC).

Fig S2

A

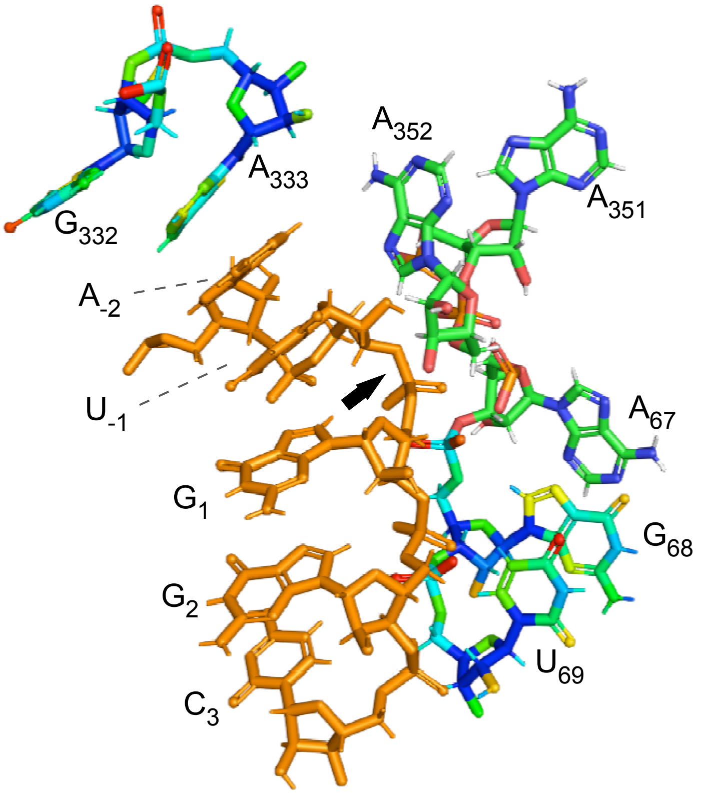

A352
A351
A333
G332
A-2
U-1
A67
G1
G68
G2
U69
C3

B

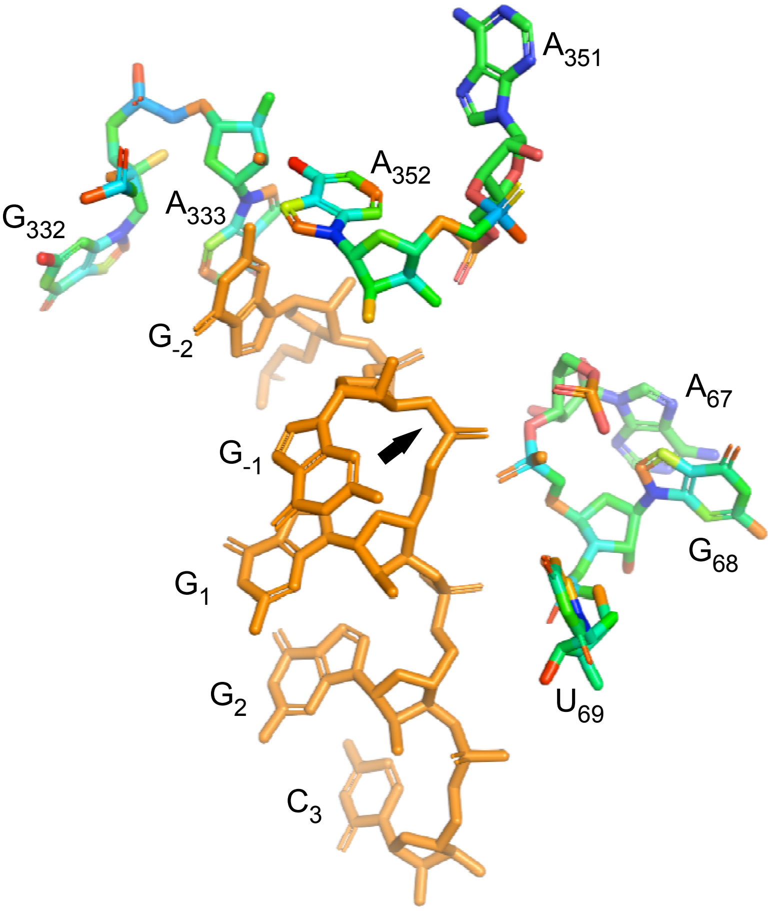

A351
A352
G332
A333
G-2
A67
G-1
G1
G68
U69
G2
C3

Fig S2

C

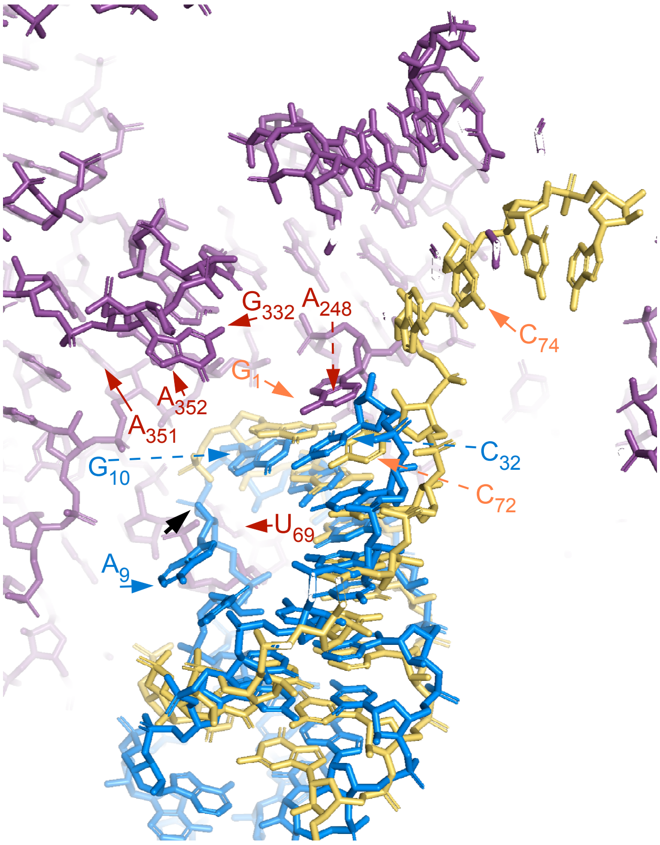
G332
A248
C74
G1
A352
A351
G10
C32
C72
U69
A9

D

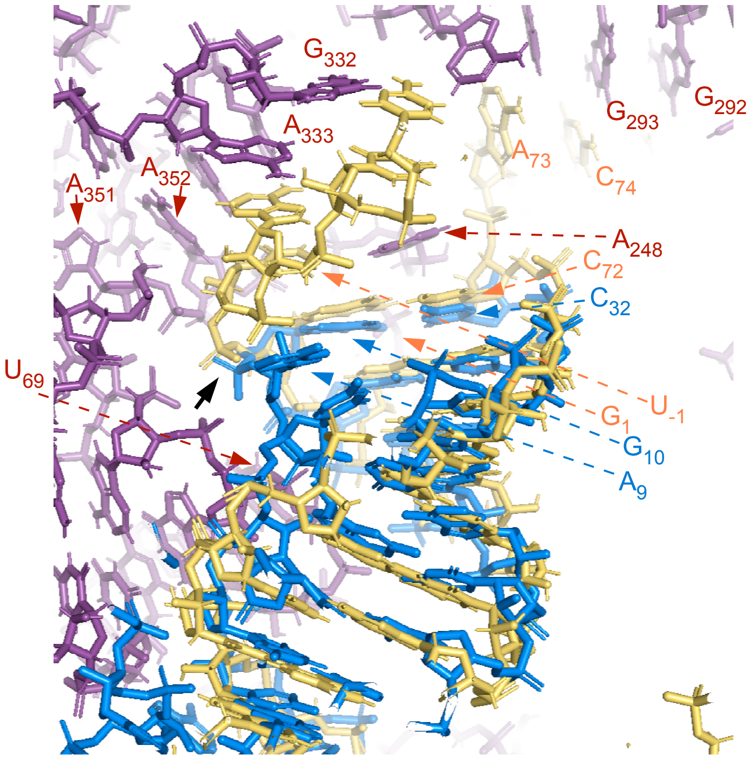
G332
G293
G292
A333
A73
C74
A351
A352
A248
C72
C32
U69
U-1
G1
G10
A9

Figure S3

(A) Modeling of the U_{332} RPR variant in complex with $pPKC_9G_{33}$ (see main text and Figure 9). U_{332} interacts with C_9 in $pPKC_9G_{33}$, favoring cleavage at the alternative site m-1 (marked with a black arrow). Pink- and cyan-coded nucleotides represent residues in the RPR and $pPKC_9G_{33}$, respectively. Green spheres mark the position of Mg^{2+} near the cleavage site. For clarity, other residues belonging to the complex were omitted.

(B) Estimated frequency of cleavage at the alternative m-1 site for the N_{332} *Eco* RPR variants using modeling data.

Fig S3

A

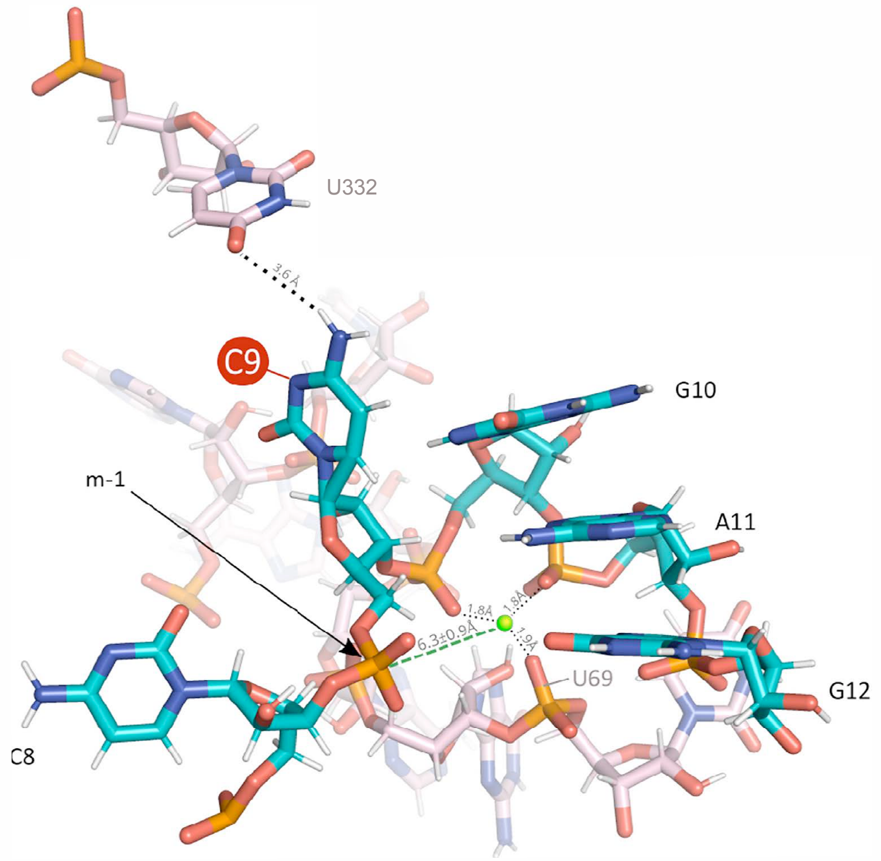

U332
3.6 Å
C9
G10
m-1
A11
1.8Å
1.8Å
6.3±0.9Å
1.9Å
U69
G12
C8

B

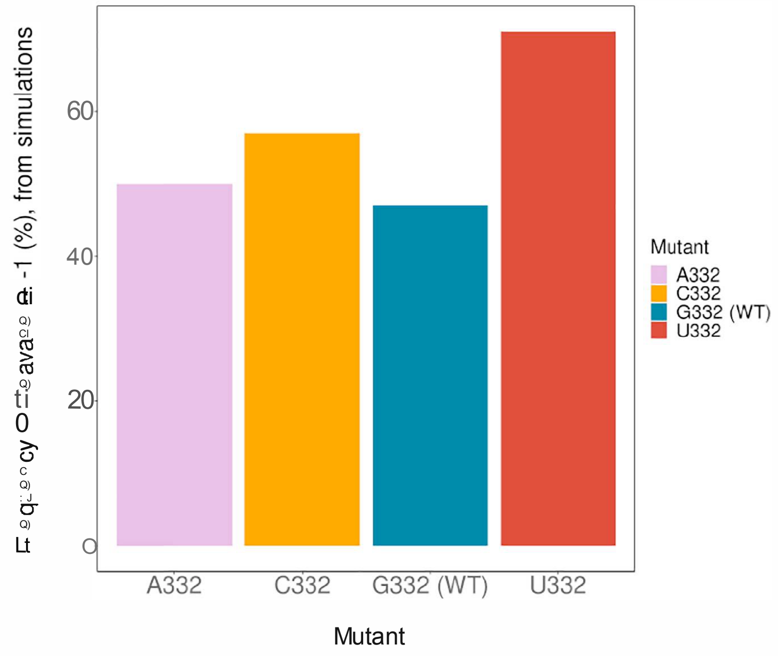

-1 (%), from simulations
0
20
40
60
A332
C332
G332 (WT)
U332
Mutant
Mutant
A332
C332
G332 (WT)
U332
